# Malaria-specific Type 1 regulatory T cells are more abundant in first pregnancies and associated with placental malaria

**DOI:** 10.1016/j.ebiom.2023.104772

**Published:** 2023-08-25

**Authors:** Adam S. Kirosingh, Alea Delmastro, Abel Kakuru, Kattria van der Ploeg, Sanchita Bhattacharya, Kathleen D. Press, Maureen Ty, Lauren de la Parte, Jimmy Kizza, Mary Muhindo, Sebastien Devachanne, Benoit Gamain, Felistas Nankya, Kenneth Musinguzi, Philip J. Rosenthal, Margaret E. Feeney, Moses Kamya, Grant Dorsey, Prasanna Jagannathan

**Affiliations:** a Stanford University School of Medicine, Stanford, USA; b Infectious Diseases Research Collaboration, Kampala, Uganda; c University of California, San Francisco, USA; d Makerere University, Kampala, Uganda; e Université Paris Cité, INSERM, BIGR, F-75014 Paris, France

**Keywords:** Malaria, Pregnancy, Gravidity, CD4^+^ T cells, Placental malaria, Immunity

## Abstract

**Background:**

Malaria in pregnancy (MIP) causes higher morbidity in primigravid compared to multigravid women; however, the correlates and mechanisms underlying this gravidity-dependent protection remain incompletely understood. We aimed to compare the cellular immune response between primigravid and multigravid women living in a malaria-endemic region and assess for correlates of protection against MIP.

**Methods:**

We characterised the second trimester cellular immune response among 203 primigravid and multigravid pregnant women enrolled in two clinical trials of chemoprevention in eastern Uganda, utilizing RNA sequencing, flow cytometry, and functional assays. We compared responses across gravidity and determined associations with parasitaemia during pregnancy and placental malaria.

**Findings:**

Using whole blood RNA sequencing, no significant differentially expressed genes were identified between primigravid (n = 12) and multigravid (n = 11) women overall (log 2(FC) > 2, FDR < 0.1). However, primigravid (n = 49) women had higher percentages of malaria-specific, non-naïve CD4^+^ T cells that co-expressed IL-10 and IFNγ compared with multigravid (n = 85) women (p = 0.000023), and higher percentages of these CD4^+^ T cells were associated with greater risks of parasitaemia in pregnancy (R_s_ = 0.49, p = 0.001) and placental malaria (p = 0.0073). These IL-10 and IFNγ co-producing CD4^+^ T cells had a genomic signature of Tr1 cells, including expression of transcription factors *cMAF* and *BATF* and cell surface makers *CTLA4* and *LAG-3*.

**Interpretation:**

Malaria-specific Tr1 cells were highly prevalent in primigravid Ugandan women, and their presence correlated with a higher risk of malaria in pregnancy. Understanding whether suppression of Tr1 cells plays a role in naturally acquired gravidity-dependent immunity may aid the development of new vaccines or treatments for MIP.

**Funding:**

This work was funded by 10.13039/100000002NIH (PO1 HD059454, U01 AI141308, U19 AI089674, U01 AI155325, U01 AI150741), the 10.13039/100000912March of Dimes (Basil O'Connor award), and the 10.13039/100000865Bill and Melinda Gates Foundation (OPP 1113682).

**Research in context**

**Evidence before this study**

In malaria-endemic settings, women who are pregnant for the first time (primigravid) are more likely to develop placental malaria (PM) compared to women who have had multiple pregnancies (multigravid). Although malaria-specific antibodies likely play an important role in mediating this gravidity-dependent protection, the role of the cellular immune response—particularly malaria-specific T cells—is less clear.

**Added Value of this study**

We conducted a comprehensive investigation of malaria-specific cellular immunity of primigravid and multigravid women from two separate cohorts in Uganda. Through this analysis, we identified a population of activated, malaria-specific, interleukin-10 (IL-10) and interferon-gamma (IFNγ) expressing CD4^+^ T cells that were more common in primigravid versus multigravid women. Transcriptomic profiling of IL-10 and IFNγ co-producing cells identified a molecular signature consistent with Type 1 regulatory (Tr1) cells, and higher percentages of these cells were associated with higher parasite prevalence during pregnancy as well as PM. In contrast, multigravid women had higher percentages of malaria-specific, tumour necrosis factor-alpha (TNFα) -expressing CD4 T cells that correlated with protection from malaria in pregnancy.

**Implications of all the available evidence**

These findings suggest an important role for Tr1 in the immune response to malaria in pregnancy and the pathogenesis of PM. Understanding the role of Tr1 cells, and TNFα expressing CD4^+^ T cells, in gravidity-dependent antimalarial protection may help efforts towards developing vaccines and other therapeutic interventions to protect pregnant women from the adverse outcomes of malaria in pregnancy.

## Introduction

Malaria during pregnancy remains a significant cause of morbidity and mortality in sub-Saharan Africa.^1^ The increased susceptibility of pregnant women to malaria is due, at least in part, to a unique physiologic niche provided by the human placenta. During pregnancy, the expression of the variant surface antigen of *Plasmodium falciparum* (*Pf*) VAR2CSA allows the parasite to bind to chondroitin sulphate A (CSA) on the placental syncytiotrophoblast, leading to placental sequestration of parasites, commonly referred to as placental malaria.^2^, ^3^, ^4^ Malaria in pregnancy can result in a number of maternal complications, such as anaemia and hypertension, and adverse perinatal outcomes, such as intrauterine growth restriction, low birth weight, foetal anaemia, and foetal mortality.^5^

The World Health Organisation recommends the use of long-lasting insecticidal bed nets (LLINs) and intermittent preventive treatment with sulfadoxine-pyrimethamine during pregnancy (IPTp-SP) to prevent infection with *Pf* parasites and reduce the risk of adverse birth outcomes. However, there is concern for diminished efficacy of these interventions due to vector resistance to pyrethroid insecticides used in LLINs and widespread parasite resistance to SP.^6^^,^^7^ Thus, there is an urgent need to develop new interventions to combat malaria during pregnancy.

With successive pregnancies, women living in malaria endemic settings develop immunity against the adverse consequences of malaria during pregnancy after repeated *Pf*-exposures. Gravidity-dependent immunity against PM has been hypothesised to be mediated by antibodies against VAR2CSA,^8^ the variant antigen expressed on the surface of *Pf*-infected erythrocytes that allows sequestration in the placenta through binding to CSA.^3^^,^^5^ Although several studies have shown parity-dependent increases in anti-VAR2CSA antibodies,^4^^,^^9^, ^10^, ^11^ not all studies have observed evidence for a protective effect of these antibodies in preventing PM and/or adverse birth outcomes. In a recent systematic review, several pregnancy-specific antibody responses were associated with a higher risk of maternal peripheral and placental infections, suggesting that these responses may be a markers of exposure rather than protection.^12^ In addition, six antibody features including opsonization, and phagocytosis of infected red blood cells (iRBCs), correlated with protection from malaria in pregnancy, but no individual pathway was sufficient for protection.^13^ Identification of additional immunologic correlates that underlie gravidity-dependent anti-malarial protection in pregnancy could therefore assist in risk stratification of pregnant patients and in development of novel malaria control strategies, including vaccines.

Malaria-specific CD4^+^ T cells could play an important role in gravidity-dependent protection given their roles in orchestrating the adaptive immune response^14^ and in immunity against blood-stage *Pf* parasitaemia. T helper (Th1) cells, which stimulate phagocytic cells to kill iRBCs, and T follicular helper (Tfh) cells, which are required to produce antigen-specific antibodies, are likely critical to protective immunity against malaria.^15^, ^16^, ^17^, ^18^ In addition, IFNγ and IL-10 co-producing cells have been shown to expand following *Plasmodium* infections in mice^19^, ^20^, ^21^, ^22^ and humans,^23^, ^24^, ^25^ and in malaria-endemic settings they are the dominant response in children with malaria.^26^ These IFNγ/IL-10 co-producing cells (also known as Type 1 regulatory T cells (Tr1)) are a Th subset with regulatory potential distinct from regulatory T cells (Tregs), and do not express the canonical Treg expression factor FoxP3.^27^ Notable transcription factors associated with Tr1 development include Blimp-1 (PRDM1) and cMAF.^25^^,^^28^ In the context of malaria in children and adults, Tr1 cells are hypothesised to prevent pathology but also to allow for parasite persistence by suppressing the Th1 response.^29^ The role of these cells in the pathogenesis of malaria during pregnancy has not been well studied. Plasma levels of IL-10 in pregnant women with malaria have been shown to be higher than those in uninfected women,^30^^,^^31^ and levels of malaria non-specific, IFNγ and IL-10 co-producing cells were elevated in pregnant versus non-pregnant women living in Papua New Guinea.^32^ A better understanding of the CD4^+^ T cell response to malaria in pregnancy could help inform the design of effective vaccines in malaria-endemic settings.

Here, we analysed the cellular immune response among primigravid and multigravid pregnant women living in eastern Uganda, a high malaria transmission setting, using a multimodal approach combining RNA sequencing, multiparameter flow cytometry, and functional assays. We compared immune responses between primigravid and multigravid women and assessed for correlates of protection against clinical malaria and PM. Our data provide insight into the shifting quality of the malaria-specific CD4^+^ T cell response across pregnancies, and we identify malaria-specific CD4^+^ T cell features that correlate with protection against malaria in pregnancy. Collectively, our data suggest that malaria-specific, IL-10 and IFNγ producing Tr1 cells are more commonly found in primigravid women and are associated with an increased risk of malaria in pregnancy. In contrast, multigravid women had higher percentages of CD4^+^ T cells that produced TNFα in the absence of IFNγ and IL-10, and these cells were correlated with protection against malaria in pregnancy.

## Methods

### Ethics

The clinical study protocols, including approval for collection and analysis of samples, were approved by the Makerere University School of Biomedical Sciences Ethics Committee, the Uganda National Council of Science and Technology, and the University of California San Francisco Committee on Human Research. The University of California San Francisco Committee on Human Research served as a single IRB for Stanford University for these study protocols. Analysis of deidentified patient samples was performed at Stanford University following IRB approval (IRB 41197). All participants provided written informed consent before enrolment.

### Clinical cohort and study procedures

Samples from pregnant women for this study were obtained from women enrolled in two clinical trials of IPTp in Tororo (NCT02163447, (PROMOTE, 2014)^27^) and Busia districts (NCT04336189, (DPSP, 2020- ongoing)). In these adjacent districts in eastern Uganda, malaria transmission is high and year-round, with two seasonal peaks.^27^^,^^33^ The trials were similar in several ways: 1) eligibility, 2) randomization to different IPTp regimens at enrolment, 3) follow-up care, 4) routine visits, and 5) analysis of placental tissue after delivery. In both studies, pregnant women were eligible for enrolment if they were between 12 and 20 weeks of gestation, 16 years or older, HIV negative, agreed to come to the study clinic for any illness, had no history of antimalarial therapy during the current pregnancy, and provided written informed consent. Women were randomized to different IPTp regimens at enrolment, and study drugs were administered beginning at 16 or 20 weeks of age and given through delivery. Women were followed for all care at a dedicated study clinic. Those presenting with fever and evidence of parasitaemia by microscopy during the study were treated with artemether-lumefantrine for symptomatic malaria as per Ugandan Ministry of Heath guidelines. Routine visits were performed every 4 weeks to assess for parasitaemia. At delivery, placental tissue was analysed for histopathologic evidence of PM using established methods.^34^ The main differences in the trials include: 1) IPTp regimens and 2) detection methods of parasitaemia. In the PROMOTE Birth Cohort 1 (PROMOTE) study, women were randomized at enrolment to receive dihydroartemisinin–piperaquine (DP) every 4 weeks, DP every 8 weeks, or SP every 8 weeks. In the DPSP trial, women were randomized to receive SP, DP, or DP + SP every 4 weeks. Detection methods of parasitaemia at routine visits and in placental blood at the time of delivery differed in the study cohorts. In the PROMOTE study, loop-mediated isothermal amplification (LAMP, Eiken Chemical) was employed to evaluate dried blood spots collected during pregnancy or at delivery for malaria parasite presence. In the DPSP study, an ultrasensitive quantitative polymerase chain reaction (qPCR) was utilised on cryopreserved whole blood during pregnancy or at delivery.^35^^,^^36^ Samples from non-pregnant women were obtained from age-matched women followed in the ongoing Program for Resistance, Immunology, Surveillance, and Modelling of Malaria in Uganda (PRISM) Border Cohort study in the Tororo and Busia districts of Uganda. The design and population of the PRISM Border Cohort study have recently been described.^33^

### Immunologic sample collection and processing

In the PROMOTE study, ∼6–10 mls of whole blood was obtained at enrolment and delivery. Plasma was collected and stored at −80°, and peripheral blood mononuclear cells (PBMC) were isolated by density gradient centrifugation (Ficoll-Histopaque; GE Life Sciences) within 4 h of blood collection and cryopreserved in liquid nitrogen. In the present study, a random selection of samples from participants in the DP every 4 weeks or SP every 8 weeks arms collected both at enrolment and delivery were used. In the DPSP study, whole blood was collected at the enrolment visit, and cryopreserved either in PAXgene® reagent or processed for plasma and PBMC isolation as above. In the present study, a random selection of samples from DPSP participants collected at enrolment were used. For non-pregnant age-matched controls followed in the PRISM Border Cohort study, whole blood collected in PAXgene® reagent and PBMCs were obtained from a random selection of subjects. Cryopreserved samples were shipped to Stanford for further analysis.

### Whole blood RNAseq

Samples collected from the DPSP study (n = 23) and PRISM Border Cohort (n = 10, controls) were analysed by whole blood RNA Sequencing, with library preparation and sequencing performed at Novogene Corporation, Inc. Sample sizes were chosen based on sample availability, cost of assays, and available literature suggesting that samples sizes of 20–30 participants would provide reasonable power to identify differentially expressed genes between groups.^37^ Briefly, whole blood samples collected in PAXgene® reagent were first treated with Proteinase K, and then RNA extraction was performed using Quick-RNA MagBead Kit (R2132) on KingFisher followed by sample quality control checks using a Qubit and Bioanalyzer 2100. Libraries were prepared using Zymo-Seq RiboFree Total RNA Library Kit (R3000). Sequencing took place on a Nova 6000 on an S4 lane, 30M paired reads, PE 150. The sequencing data were uploaded to the Galaxy web platform (usegalaxy.org), where we performed quality control, groomed with FASTQ groomer, aligned to hg38 with bowtie 2, filtered using SAM Tools and calculated gene expression using featureCounts.^38^, ^39^, ^40^ We have included a supplemental table (Supplemental Table S1) which presents RNA quality control metrics including concentration and volume for each sample analysed in this study.

### T cell phenotyping

T cell phenotyping was performed utilizing all available PROMOTE (n = 99) PBMC samples. Thawed PBMCs were rested for 12 h at 37 °C in complete RPMI (RPMI (Corning) supplemented with 10% FBS (Gibco), 100 IU Penicillin (Corning), 100 μg/mL Streptomycin (Corning), 1 mM Hepes (Corning) and 2 mM l-glutamine (Corning). The following morning, PBMC were washed, and surface stained with CXCR5 BV711 (BioLegend, Clone: J252D4, RRID: AB_2629526, 1:50 dilution) for 30 min at 37 °C. PBMCs were then stained with remaining antibodies for 30 min at room temperature (RT) in the dark. The surface-labelled antibodies used were: anti-CXCR3 BV421 (BD Biosciences, clone IC6 RRID:AB_2737653, 1:10 dilution), LIVE/DEAD Aqua (Invitrogen, Cat# L34965, 1:200 dilution), anti-CD14 BV510 (BioLegend, Clone:M5E2, RRID:AB_2561946, 1:100 dilution), anti-CD19 BV510 (BioLegend, Clone:HB19, RRID:AB_2561668, 1:100 dilution), anti-CD25 BV605 (BD Biosciences, Clone:2A3, RRID:AB_2744343, 1:25 dilution), Clone:B56, RRID:AB_396302, 1:20 dilution), anti-PD-1 PE (BD Biosciences, Clone:EH12.1, RRID:AB_2033989, 1:20 dilution), anti-CD4 PerCP (BioLegend, Clone:RPA-T4, RRID:AB_893321, 1:50 dilution), anti-CCR6 APC-R700 (BD Biosciences, Clone:11A9, RRID:AB_2739092, 1:16.5 dilution), anti-ICOS APC-Cy7 (BioLegend, Clone:C398.4A, RRID:AB_2566128,1:20 dilution). Cells were washed twice with PBS containing 0.5% BSA and 2 mM EDTA before acquisition on an Attune NXT flow cytometer.

### Parasite culture and isolation for functional assays

VAR2CSA-expressing CS2 *Pf*^41^ parasites were obtained through BEI Resources (NIAID, NIH: Plasmodium falciparum, Strain CS2, MRA-96, contributed by Stephen J. Rogerson.) CS2 parasites were cultured in RPMI 1640 with 25 mM HEPES, 25 mM sodium bicarbonate, 1% gentamycin, and enriched with 0.5% Albumax (pH 6.75) and 250uM hypoxanthine, under atmospheric conditions (5% oxygen, 5% carbon dioxide, and 95% nitrogen). To retain synchronous cultures, parasite growth was treated with 5% D-sorbitol. Schizont isolation was performed at 5–10% parasitaemia using MACS cell separation LD columns (Miltenyi Biotec) and cryopreserved in glycerolyte prior to use. For functional experiments, schizonts were thawed, washed, and resuspended in RPMI before addition to cells. Mycoplasma contamination was assayed using the MycoAlert Mycoplasma Detection Kit (Lonza).

### Activation induced marker assay

For the activation induced marker (AIM) assay, a random sampling of PBMCs were analysed from primigravid (n = 21) and multigravid (n = 23) pregnant women enrolled in the DPSP study. Sample size was ascertained according to sample availability and published reports.^42^ Briefly, thawed PBMCs were prepared in 96-well U-bottom plated at 1 × 10ˆ6 cells in 200 μL of complete RPMI and rested for 1 h at 37 °C. PBMCs were stimulated for 18 h in the presence of CS2 *Pf*-iRBCs at a E:T ratio (PBMC to iRBCs) of 2:1, with phytohemagglutinin (PHA) (2 μg/mL) as a positive control, or media only as a negative control.^42^ After stimulation, cells were washed and surface stained for CXCR5 BV711 (BioLegend, Clone: J252D4, RRID: AB_2629526, 1:50 dilution) for 30 min at 37 °C. This was followed by the remaining surface stain for 30 min at RT in the dark. Surface antibodies used were: anti-PD-1 BV421 (BioLegend, Clone:EH12.2H7, RRID:AB_10960742, 1:25 dilution), anti-CD14 BV510 (BioLegend, Clone:M5E2, RRID:AB_2561946, 1:100 dilution), anti-CD19 BV510 (BioLegend, Clone:HB19, RRID:AB_2561668, 1:100 dilution), anti-CD45RA BV605 (BioLegend, Clone:HI100, RRID:AB_2563814, 1:125 dilution), anti-CD4 BV650 (BioLegend, Clone:RPA-T4, RRID:AB_2632791, 1:50 dilution), anti-CD8A BV785 (BioLegend, Clone:RPA-T8, RRID:AB_2563264, 1:50 dilution), anti-CD69 FITC (BD Biosciences, Clone:FN50, RRID:AB_395915,1:40 dilution), anti-OX40 PE (BioLegend, Clone:ACT35, RRID:AB_10645478, 1:20 dilution), anti-CD137 APC (BioLegend, Clone:4B4-1, RRID:AB_830671, 1:20 dilution), anti-CD3 AF700 (BD Biosciences, Clone:SK7, RRID:AB_2563419, 1:50 dilution), anti-ICOS APC-Cy7 (BioLegend, Clone:C398.4A, RRID:AB_2566128, 1:62.5 dilution) and LIVE/DEAD Aqua (Invitrogen, Cat# L34965, 1:200 dilution). Cells were washed twice with PBS containing 0.5% BSA and 2 mM EDTA before acquisition on an Attune NXT flow cytometer.

### Intracellular cytokine staining assay

For intracellular cytokine staining (ICS) assays, all available PBMC samples from PROMOTE (n = 90) were analysed. Randomly selected samples from DPSP (n = 43) were also analysed by ICS as both validation of PROMOTE results and to assess responses to SARS-CoV-2 peptides. Sample sizes were chosen according to sample availability and our prior studies detailing malaria-specific CD4^+^ T cell percentages.^26^ After thawing, PBMCs were prepared in 96-well plates with 1 × 10ˆ6 cells in 200 μL of complete RPMI then rested for 1 h at 37 °C in a CO2 incubator. PBMCs were stimulated with media only, iRBCs (24 h, E:T ratio of 2:1), phorbol myristate acetate/ionomycin (PMA/Io) (4 h), or PepTivator® SARS-CoV-2 lyophilized peptide pools from Miltenyi Biotec (6 h). For the spike ‘S’ glycoprotein of SARS-CoV-2 we used the combination of Prot_S1 and Prot_S peptide pools, which covers the aa sequence 1–692 of the N-terminal S1 domain (GenBank: MN908947.3, Protein QHD43416.1) and the immunodominant sequence domains, within aa 304–1273, of the S protein (GenBank: MN908947.3, Protein QHD43416.1), respectively. Brefeldin A (BD Pharmingen) and Monensin (BD Pharmingen) were added 6 h after stimulation by iRBC and immediately with PMA/Io and S peptides. Following incubation cells were washed with PBS containing 0.5% BSA and 2 mM EDTA and anti-CCR7 BV 421 (BioLegend, Clone:G043H7, RRID:AB_11203894, 1:20 dilution) was added directly to the cell pellet and incubated for 30 min at 37 °C followed by the remaining surface stain for 30 min at RT in the dark. Cells were then fixed/permeabilized (FIX & PERM ® Cell Permeabilization Kit, Invitrogen) and stained with intracellular antibodies for 30 min RT. Antibodies used were: LIVE/DEAD Aqua (Invitrogen, Cat# L34965, 1:200 dilution), anti-CD14 BV510 (BioLegend, Clone:M5E2, RRID:AB_2561946, 1:100 dilution), anti-CD19 BV510 (BioLegend, Clone:HB19, RRID:AB_2561668, 1:100 dilution), anti-CD45RA BV605 (BioLegend, Clone:HI100, RRID:AB_2563814, 1:125 dilution), anti-CD4 BV650 (BioLegend, Clone:RPA-T4, RRID:AB_2632791, 1:50 dilution), anti-CD8A BV785 (BioLegend, Clone:RPA-T8, RRID:AB_2563264, 1:50 dilution), anti-CXCR5 BV711 (BioLegend, Clone:J252D4, RRID:AB_2629526, 1:50 dilution), anti-Vd2 FITC (BioLegend, Clone:B6, RRID:AB_1089230, 1:50 dilution), anti-IL-10 PE (BD Biosciences, Clone: JES3-19F1, RRID:AB_397227, 1:6.25 dilution), anti-IFNγ PerCP Cy5.5 (BioLegend, Clone:4S.B3, RRID:AB_961355, 1:25 dilution), anti-IL-21 eFluor 660 (eBioscience, Clone:eBio3A3-N2, RRID:AB_10598202 1:12.5 dilution), anti-TNFα AF700 (BD Biosciences, Clone:Mab 11, RRID:AB_396978, 1:50 dilution), anti-CD3 APC-H7 (BD Biosciences, Clone:SK7, RRID:AB_1645475, 1:50 dilution). All samples were acquired on an Attune NXT flow cytometer.

### Cytokine capture and sorting

For cytokine capture experiments, PBMCs from n = 3 primigravid women enrolled in the DPSP study were utilized to maximize collection of malaria-specific IL-10 and TNFα producing subsets. Thawed PBMCs were rested for 8 h at 37 °C in a CO2 incubator at 1 × 10ˆ6 cells per well in a U-shaped 96-well plate in complete RPMI. PBMCs were then stimulated with iRBC E:T ratio of 2:1 for 12 h at 37 °C. IFN-γ^+^IL-10^+^TNFα^−^ and IFN-γ^-^IL-10^−^TNFα^+^ cells were isolated using IFN-γ Secretion Assay–Detection Kit (FITC), human (Cat# 130-090-433) TNF-α Secretion Assay–Detection Kit (PE), human (Cat# 130-091-268), and IL-10 Secretion Assay–Detection Kit (APC), human (Cat# 130-090-761), according to the manufacturer's instructions. Following staining with the detection antibody the PBMCs were surface stained for 30 min at RT with the following antibodies: anti-CD3 APC-H7 (BD Biosciences, Clone:SK7, RRID:AB_1645475, 1:50 dilution), anti-CD45RA BV605 (BioLegend, Clone:HI100, RRID:AB_2563814, 1:125 dilution), anti-CD4 BV650 (BioLegend, Clone:RPA-T4, RRID:AB_2632791, 1:50 dilution), anti-CD8A BV785 (BioLegend, Clone:RPA-T8, RRID:AB_2563264, 1:50 dilution), LIVE/DEAD Aqua (Invitrogen, Cat# L34965, 1:200 dilution), anti-CD14 BV510 (BioLegend, Clone:M5E2, RRID:AB_2561946, 1:100 dilution), anti-CD19 BV510 (BioLegend, Clone:HB19, RRID:AB_2561668, 1:100 dilution). IL-10^+^IFNγ^+^TNFα- and IL-10^−^IFNγ^-^TNFα^+^ CD4^+^ cells were sorted using FACSAria Fusion sorter.

### mRNAseq for transcriptome profiling

Approximately 300 human CD4 IL-10^+^IFNγ^+^TNFα^−^ and IL-10^−^IFNγ^-^TNFα^+^ cells were sorted directly to 10.5 μl of the 1x lysis buffer and immediately stored at −80 °C for further processing. First-strand cDNA was primed by the 3′ SMART-Seq CDS Primer II A and used the SMART-Seq v4 Oligonucleotide for template switching at the 5′ end of the transcript. Full length of cDNA was synthesized and amplified by LD PCR for 14 cycles using a SMARTseq v4 Ultra Low Input RNA kit (Cat#634888) followed by the Illumina Nextera XT library preparation kit per the manufacturer's protocol. An equal molar amount of fragmented cDNA library from each sample was pooled for sequencing on the Illumina NextSeq 550 platform at Stanford University Pathology MP core. FASTQ files were generated using the bcl2fastq2 Conversion v2.19 tool. Each set of samples seq data was aligned and normalized using STAR aligner to assemble the mapped reads into homo sapiens transcriptome hg10 Refseq.

### anti-VAR2CSA antibody assays

Primigravid (n = 22) and multigravid (n = 34) plasma collected at enrolment from DPSP participants was screened for VAR2CSA reactivity using a direct binding ELISA, which was adapted from a previous study.^43^ 96 well U-shaped plates were coated with the PRIMVAC derived VAR2CSA vaccine candidate at 1 μg/mL in 100 μL/well of PBS for 12 h at 4 °C. To generate a standard curve two rows were coated with anti-Kappa (Southern Biotech, Clone:SB81a, RRID: AB_2796674, 1.5 μg/mL) and anti-Lambda antibodies (Southern Biotech, Clone: JDC-12, RRID: AB_2796705, 1.5 μg/mL). The plates were blocked with 100 μL/well of PBS containing 4% BSA for 1 h at 37 °C. Plasma diluted to 1:100 in PBS containing 2% BSA were added to wells at a volume of 100 μL and incubated for 1 h at 37 °C. For the standard curve, a known concentration of antibody was serially diluted across the plate. Plates were washed three times with 250 μL PBS containing 0.5% Tween 20. Then 100 μL/well of AP tagged anti-human IgG (Thermo Fisher Scientific, Clone: HP6017, RRID AB_2532923, 1:4000 dilution) in PBS containing 2% BSA was incubated for 1 h at 37 °C. The plates were washed three times with 250 μL of PBS containing 0.5% Tween 20. 100 μL/well of PNPP substrate (Thermo Fisher Scientific) was added and the plates were read at 475 nm. Standard curves were fit with a linear regression and levels of VAR2CSA specific antibodies were calculated.

### Statistics

All statistical analyses were performed using STATA (version 16) (StataCrop LLC), SPICE (version 6.1) (NIAID),^44^ and/or RStudio (version 1.3.1093) (RStudio). Detailed statistical parameters are reported in the figure legends. Pie charts were generated using SPICE, heatmaps, volcano plots, bar charts, and association scatterplots were generated by using ggplot2 and tidyverse^45^ (version 2.0.0) packages in RStudio.

For RNA Sequencing, transcriptomes were compared between pregnant (DPSP) and non-pregnant (PRISM Border Cohort) participants, and primigravid vs. multigravid participants (DPSP). Preliminary analysis and visualization of gene expression counts were performed using iDEP.96.^46^ Lowly expressed genes were removed, and expression levels were normalized using variance stabilization transformation with DESeq2 (version 1.38.3), R (version 4.2.0) for downstream analysis.^47^ Differential gene expression was determined using DESeq2 with the following criteria: adjusted p-value (Benjamini Hochberg) ≤ 0.1 and absolute log 2 fold change >2. Enrichment analysis for specific gene ontology terms and pathways was performed using enrichR.^48^^,^^49^ p-values shown are calculated using a Fisher's exact test and corrected using Benjamini Hochberg.^50^ Transcriptomes from sorted PBMCs from primigravid mothers were analysed using the same pipeline. The genomic data supporting these analyses have been deposited in Gene Expression Omnibus GSE234585 (Whole Blood RNA-seq) and GSE234586 (PBMCs RNA-seq).

Flow cytometric analyses was performed using FlowJo 10 software (v10.8.0) (BD). Samples with poor or no viability (determined by Live/Dead Aqua stain (Invitrogen)) were excluded during analysis. Percentages of antigen-specific activation induced marker and cytokine producing T cells (alone or in combination) are reported after background subtraction of the percentage of the identically gated population of cells from the same sample stimulated with media control. Background subtracted responses were considered positive if >0.01% parent population. Malaria-specific cytokine production was considered both as the percentage of cytokine-producing cells among the total non-naïve CD4^+^ (nnCD4^+^) T cell population (defined as CD4^+^ T cell magnitude), as well as the percentage of cytokine-producing cells as a fraction of total cytokine-producing CD4^+^ T cells (defined as relative proportion, or percentage of total malaria-specific response^26^^,^^51^).

Comparisons of cellular percentages and antibody levels between groups was performed using the non-parametric Mann Whitney U test. The Wilcoxon matched pairs test was used to compare paired data from enrolment and delivery. Statistical analyses of global cytokine profiles (pie charts) were performed by partial permutation tests using SPICE. Continuous variables were compared using Spearman correlation with Benjamini-Hochberg^50^ multiple comparison correction.

To determine associations between gravidity (exposure) and CD4^+^ T cell measurements (outcomes), univariable linear regression models were utilized after log transformation of non-normally distributed CD4^+^ T cell percentages, which resulted in approximately normal variable distributions. For variables identified as significant on univariable analysis (p < 0.05), multivariable linear regression models were utilized to adjust for potential confounders, including maternal age and parasitaemia status at the time of sampling (directed acyclic graph shown in Supplemental Figure S1).

For analysis of associations between CD4^+^ T cell parameters measured at enrolment (exposure) and pregnancy outcomes, including *Pf* parasite prevalence during pregnancy (continuous), and placental malaria at delivery (dichotomous), both univariable and multivariable models were utilized as above, with non-normally distributed CD4^+^ T cell percentages log transformed resulting in approximately normal variable distributions. Multivariable linear and logistic regression models were adjusted for potential confounders, including gravidity, maternal age, and parasitaemia status at the time of sampling (Supplemental Figure S1). Two-sided p-values were calculated for all test statistics and p < 0.05 was considered significant.

### Role of funders

The funders did not play a role in the study design; data collection, analyses, and interpretation; manuscript preparation; and in the decision to submit the manuscript for publication.

## Results

### Cohort characteristics and burden of malaria in pregnancy among primigravid and multigravid women

To study pregnancy-specific malarial immunity and compare differences across gravidity, we analysed samples collected from pregnant women enrolled in two cohorts in adjacent malaria-endemic districts in eastern Uganda, PROMOTE (Tororo District, n = 113) and DPSP (Busia District, n = 92). In both cohorts, peripheral blood samples were collected at the enrolment visit during the second trimester (12–16 weeks gestation). For the samples selected as part of this study, the mean age of primigravid women enrolled was 19 years of age, with multigravid women older, as expected (Table 1). Parasite prevalence at enrolment was higher in primigravid versus multigravid women in the two cohorts (Table 1).

**Table 1:** Cohort characteristics.

	Cohort 1 (PROMOTE)			Cohort 2 (DPSP)		
	Primigravid (N = 34)	Multigravid (N = 79)	p-value	Primigravid (N = 41)	Multigravid (N = 49)	p-value
Age at Enrolment, Mean (SD)	18.6 (1.6)	23.7 (3.6)	<0.001	18.8 (2.1)	26.0 (5.0)	<0.001
Parasitaemia at enrolment by blood smear, n/N (%)	24/34 (70.6%)	24/79 (30.4%)	<0.001	23/44 (51.2%)	9/49 (18.4%)	0.001
Parasitaemia at enrolment by LAMP (PROMOTE) or qPCR (DPSP), n/N (%)	25/34 (73.5%)	40/79 (50.6%)	0.02	29/43 (67.4%)	28/49 (57.1%)	0.31
Maternal IPTp arm (PROMOTE only)^a^						
SP every 8 weeks, n/N(%)	18 (53%)	37 (47%)	0.48	n/a	n/a	
DP every 4 weeks, n/N(%)	16 (47%)	42 (53%)		n/a	n/a	
Symptomatic malaria incidence during pregnancy, episodes per person year	0.69	0.53	0.51	0.55	0.08	0.015
Parasite prevalence during pregnancy,^b^ n/N (%)	104/239 (43.5%)	142/561 (25.3%)	0.002	61/251 (24.3%)	73/226 (24.4%)	0.98
Placental malaria at delivery by histopathology, n/N (%)	22/33 (66.6%)	16/79 (20.3%)	<0.001	25/40 (62.5%)	8/44 (18.2%)	<0.001
Placental malaria at delivery by parasite detection (LAMP (PROMOTE) or qPCR (DPSP)), n/N (%)	5/32 (15.6%)	1/79 (1.3%)	0.002	1/36 (2.8%)	3/43 (7.0%)	0.40

IQR: inner quartile range; IPTp: Intermittent preventive therapy in pregnancy; SP: sulfadoxine pyrimethamine; DP: dihydroartemisinin-piperaquine; LAMP: loop-mediated isothermal amplification; qPCR: quantitative PCR.

a IPTp assignments not available for DPSP trial since it is ongoing.

b Parasite prevalence during pregnancy measured by LAMP (PROMOTE) or qPCR (DPSP).

During pregnancy, parasite prevalence measured at the time of routine visits and at the time of delivery was significantly higher among primigravid women compared to multigravid women in the PROMOTE study (Table 1); although parasite prevalence was similar between multigravid and primigravid women in the DPSP study, 2/3 of women in this study are receiving IPTp with DP every 4 weeks, which we have previously shown to significantly reduce parasitaemia in pregnancy.^52^^,^^53^ In the two cohorts, at the time of delivery, more than 60% of primigravid women versus less than 25% multigravid women had evidence of PM by histopathology (Table 1). Next, we compared levels of VAR2CSA-specific IgG in these women. Among women who were aparasitaemic, VAR2CSA-specific IgG levels at enrolment were higher among multigravid compared to primigravid women (Supplemental Figure S2a) while levels where similar among primigravid and multigravid women with parasitaemia (Supplemental Figure S2b).

### Whole blood transcriptomics reveal differential expression of TSLP among pregnant primigravid women with parasitaemia

**Fig. 1:**
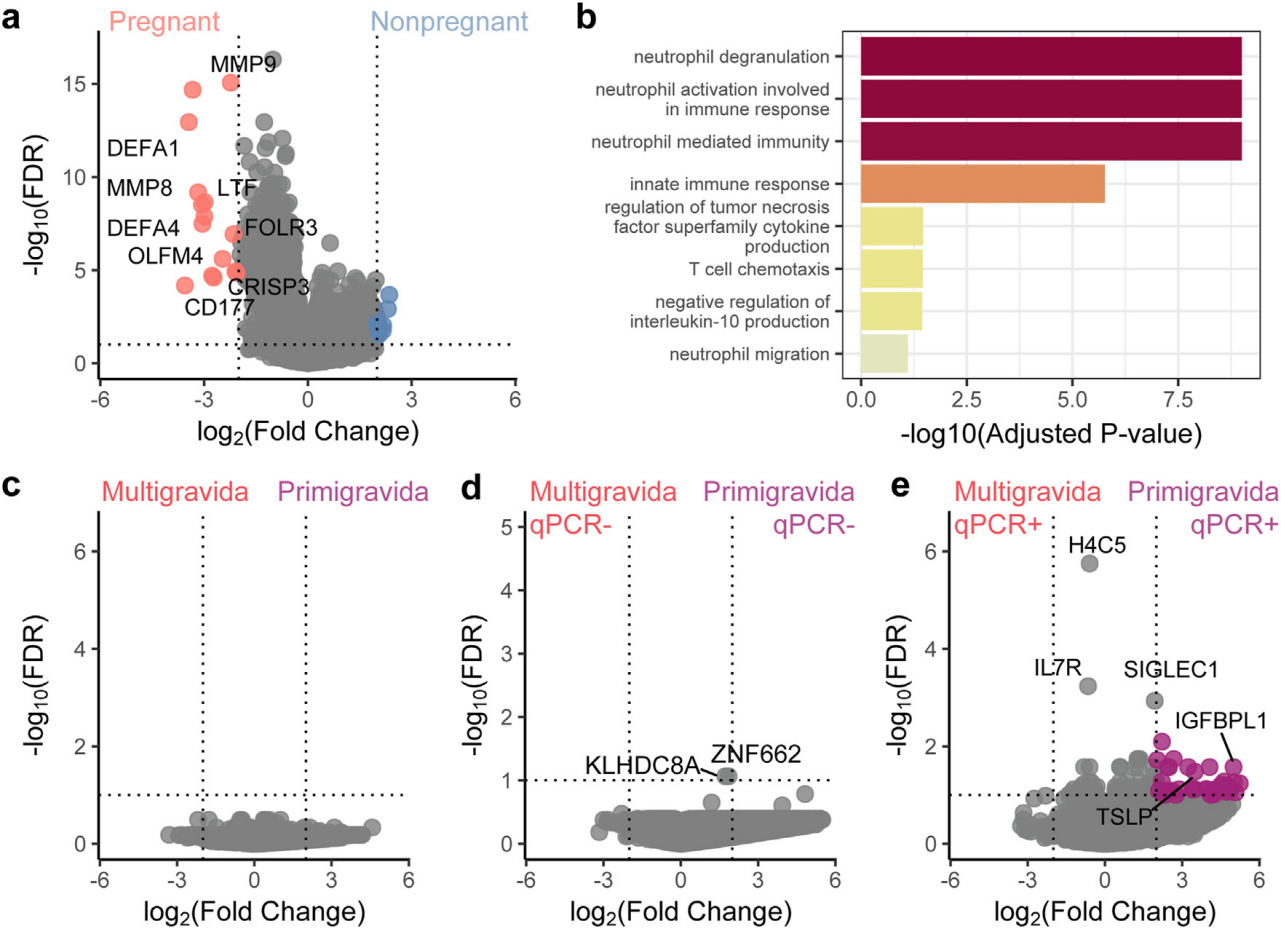
**Whole blood transcriptomic profiling of malaria during pregnancy reveals differences between pregnant and non-pregnant women as well as qPCR** + **primigravid and qPCR** + **multigravid women.** (a) Differentially expressed genes (DEGs) of non-pregnant women (n = 10, blue) to pregnant women (n = 23, red) depicted in a volcano plot. (b) Selected gene expression pathways upregulated in pregnant women compared to non-pregnant women in a malaria endemic setting. (c) DEGs of primigravid women (n = 12) compared to multigravid women (n = 11), (d) qPCR-primigravid women (n = 4, purple) compared to qPCR-multigravid women (n = 6), and (e) qPCR + primigravid women (n = 8, purple) compared to qPCR + multigravid women (n = 5). DEGs had an adjusted p-value (Benjamini Hochberg) < 0.1 and minimum log 2-fold change of 2. Selected genes were annotated in volcano plots.

To broadly analyse the cellular immune response in pregnancy among women in a malaria-endemic setting, and compare responses across gravidity, we performed whole blood RNA sequencing on peripheral blood taken at enrolment. We first compared responses between age-matched (17–24 years old) pregnant (DPSP, n = 23) and non-pregnant women enrolled in a contemporary cohort in the same Ugandan districts (n = 10, PRISM Border Cohort study). Overall, 24 differentially expressed genes (DEGs) were found when comparing pregnant versus non-pregnant women (log 2(FC) > 2, FDR < 0.1, Fig. 1a, Supplemental Table S2). Neutrophil degranulation and activation [defensin peptide *DEFA1*, transferrin *LTF*, glycoprotein *CD177*, folate receptor gamma *FOLR3*], T cell chemotaxis [defensin peptide *DEFA4*] and cytokine production of TNF and IL-10 [matrix metallopeptidase 8 *MMP8* ] were among the pathways upregulated in pregnant women, regardless of parasitaemia status (Supplemental Figure S3a and b), consistent with prior reports comparing the transcriptional state of healthy pregnant and non-pregnant individuals^54^, ^55^, ^56^ (Fig. 1b, Supplemental Table S3).

When comparing whole blood gene expression between primigravid pregnant women (n = 12) and multigravid pregnant women (n = 11) (Fig. 1c) and when considering only women without parasitaemia (by qPCR) (Fig. 1d) no significant DEGs overall were found between the groups (log 2(FC) > 2, FDR < 0.1). When analysing *Pf* qPCR + women only, 36 genes were highly expressed in primigravid compared with multigravid women (Fig. 1e, Supplemental Table S4). Among them was Thymic Stomal Lymphopoietin (*TSLP*), which is expressed by multiple cell types, including cytotrophoblasts, which have been described to circulate during pregnancy.^57^ *TSLP* has been shown to play an important role in regulatory CD4^+^ T cell differentiation via polarization of dendritic cells.^58^ In addition, *SIGLEC1*, an immunoregulatory marker in viral infections,^59^ was more highly expressed in primigravid women. No significant DEGs were found when comparing parasitemic women versus aparasitaemic pregnant women (Supplemental Figure S3c). Together these data suggest that, while the whole blood transcriptional response is similar between primigravid and multigravid women, it differs among primigravid and multigravid women with parasitaemia.

### Primigravid women have higher percentages of malaria-specific activated CD4^+^ T cells than multigravid women

Given the higher gene expression of *TSLP* observed in parasitemic, primigravid women, and the documented role of *TSLP* in CD4^+^ T cell differentiation,^58^ we next investigated differences in malaria-specific CD4^+^ T cell subsets and response. Overall, percentages of CD4^+^ T cells, FoxP3^+^ Tregs, CXCR5^+^ PD1^+^ Tfh cells, and Tfh subsets (Th1, Th2, and Th17) were similar by flow cytometry between primigravid and multigravid women (Supplemental Figure S4, Supplemental Figure S5).

**Fig. 2:**
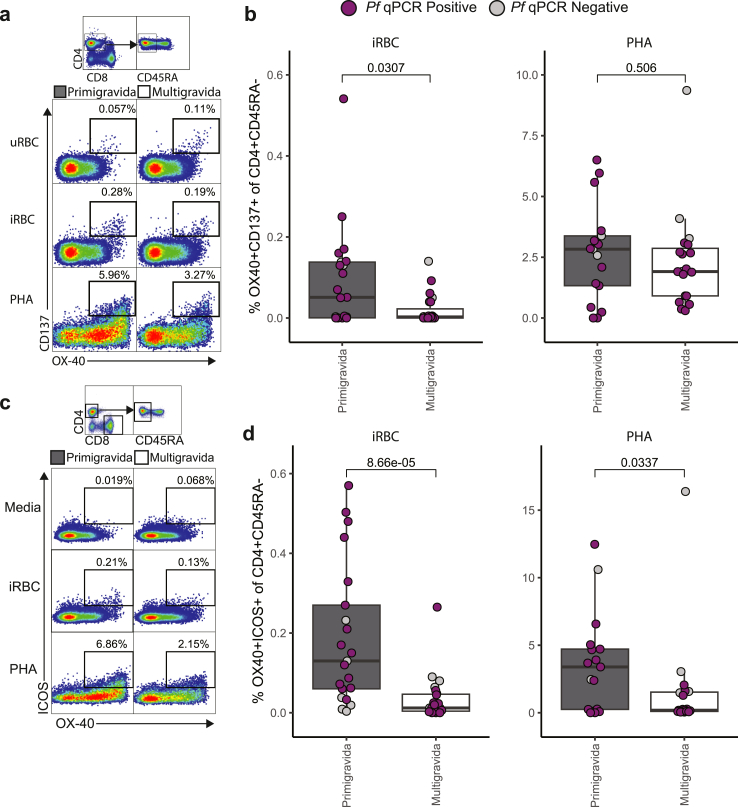
**Activation induced marker assay comparing activated malaria-specific CD4**^**+**^**T cells among primigravid and multigravid women**. Representative dot plots of activated malaria-specific CD45RA^−^ CD4^+^ T cells expressing OX40 and (a) CD137 or (c) ICOS stimulated with media only (negative control), *Pf*-iRBCs, or PHA. Percentage of CD45RA^−^ CD4^+^ T cells expressing OX40 and (b) CD137 or (d) ICOS of primigravid (dark grey, iRBC n = 21, PHA (positive control) n = 17) and multigravid (white, iRBC n = 23, PHA n = 19) stimulated by iRBC (left) and PHA (right) (purple = *Pf* qPCR positive, light grey = *Pf* qPCR negative, dark grey = untested). Shown are box and whiskers plots with hinges at 25% and 75% and whiskers extended by the interquartile range multiplied by 1.5. p-values are calculated using a Mann Whitney U test.

To investigate malaria-specific CD4^+^ T cell function, we first used an activation-induced marker (AIM) among participants enrolled in the DPSP study. To measure malaria-specific CD4+ T cell activation, we stimulated PBMCs with VAR2CSA expressing CS2 *Pf*-iRBCs, PHA, or media, and analysed the expression of OX40 and CD137 (Fig. 2a). These activation markers are critical for T cell survival and proliferation.^60^ In addition we analysed inducible T cell costimulator (ICOS), which regulates cytokine expression.^61^ Primigravid women had significantly higher percentages of OX40^+^ CD137^+^ (AIM^+^) CD45RA^−^ CD4^+^ T cells following iRBC stimulation compared with multigravid women despite similar responses to polyclonal stimulation (Fig. 2a and b). Both associations were independent of parasitaemia at the time of sampling (Supplemental Figure S6) or participant age (Supplemental Figure S7). We observed a significantly higher population of ICOS + OX40+ CD45RA- CD4+ T cells in primigravid women when stimulated with iRBC or PHA (Fig. 2c and d), although iRBC responses strongly correlated with responses to PHA (R_s_ = 0.71, p = 0.0000012 [spearman correlation]). These findings suggest that ICOS + OX40+ responses to PHA may reflect Pf-specific responses. The percentage of malaria-specific AIM^+^ Tfh cells was also higher in primigravid women compared to multigravid women (Supplemental Figure S8a and b). None of the malaria-specific activated T cell subsets correlated with VAR2CSA specific antibodies measured at concurrent timepoints (Supplemental Figure S8c, d and e). No significant differences were observed in percentages of OX40^+^ICOS^+^ CD8^+^ T cells or activated CD8^+^ (CD137^+^CD69^+^) T cells, between primigravid and multigravid women (Supplemental Figure S9a and b). Together, these data suggest that percentages of malaria-specific AIM^+^ CD4^+^ T cells, AIM^+^ Tfh cells, as well as OX40^+^ICOS^+^ cells, are higher in primigravid compared with multigravid women.

### Malaria-specific IL-10^+^ and TNFα^+^ CD4^+^ T cells exhibit differential associations with gravidity and malaria in pregnancy

**Fig. 3:**
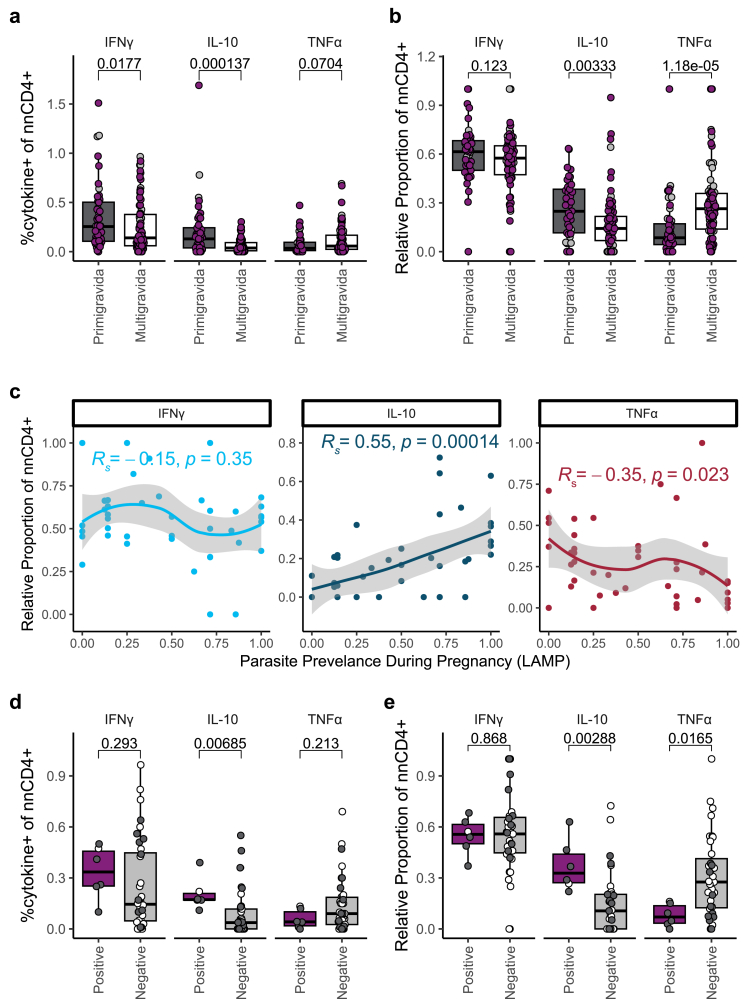
**Intracellular cytokine production of nnCD4**^**+**^**T cells differ across gravidity, parasitaemia and placental malaria outcome**. (a) The percentage of nnCD4^+^, and (b) relative proportion among total cytokine-producing CD4^+^ T cells, of nnCD4^+^ producing IFNγ, IL-10, and TNFα after 24-h stimulation with iRBCs from primigravid (n = 49, dark grey) and multigravid (n = 85, white) (purple = *Plasmodium falciparum (Pf)* LAMP positive, grey = *Pf* LAMP negative) pregnant women in PROMOTE and DPSP as measured by flow cytometry. (c) Correlations between relative proportion of nnCD4^+^ producing IFNγ, IL-10, and TNFα to iRBC stimulation with *Pf* parasite prevalence during pregnancy detected by LAMP among women receiving SP chemoprevention (n = 43, PROMOTE). Locally estimated scatterplot smoothing (LOESS) were fit to the relative proportion of nnCD4^+^ expression IFNγ (light blue), IL-10 (dark blue), and TNFα (dark red). The p-values and R-values were calculated using Spearman correlations. (d) The percentage of nnCD4^+^ (e) and relative proportion among total cytokine-producing CD4^+^ T cells, of nnCD4^+^ producing IFNγ, IL-10, and TNFα after a 24 h stimulation with iRBCs, stratified by placental malaria status as measured by placental blood LAMP *Pf* positive (n = 6, purple) and negative (n = 37, grey) among PROMOTE women given SP chemoprevention. Data shown are background (Media) subtracted. p-values are calculated by Mann Whitney U test.

To further characterise the functional status of these malaria-specific CD4^+^ T cells, we performed an intracellular cytokine staining assay following a 24-h *in vitro* stimulation with iRBCs, PMA/Ionomycin, or media, using PBMCs from both the PROMOTE and DPSP cohorts. We analysed cytokine production among non-naïve CD4^+^ (nnCD4^+^) T cells, which were identified by excluding CCR7^+^CD45RA^+^ CD4^+^ T cells (Supplemental Figure S4), and quantified percentages of CD4^+^ T cells expressing inflammatory (IFNγ, TNFα) and anti-inflammatory (IL-10) cytokines. Primigravid women had higher percentages of IL-10 producing nnCD4^+^ T cells in the two cohorts combined (p = 0.000137, Fig. 3a [Mann Whitney U test]), and when compared in the cohorts separately (p = 0.0172 in DPSP, p = 0.00367 in PROMOTE (Supplemental Figure S10) [Mann Whitney U test]), and this association remained significant after adjusting for maternal age and parasitaemia status at the time of sampling (Supplemental Table S5). While the percentage of IFNγ-expressing nnCD4^+^ T cells was significantly higher in primigravid women in both cohorts combined (p = 0.0177, Fig. 3a [Mann Whitney U test]), these differences, along with a trend of higher TNFα in multigravid women, were not significant in the PROMOTE cohort alone nor after adjusting for maternal age and parasitaemia status (Supplemental Table S5). When considering the proportion of each cytokine with respect to the total malaria-specific cytokine-producing response, the relative proportion of TNFα cytokine producing cells was significantly higher in multigravid compared to primigravid women in both cohorts combined (p = 0.000012 (Fig. 3b) [Mann Whitney U test]) and in each individual cohort (Supplemental Figure S10), and this remained significant after adjusting for maternal age and parasitaemia status at the time of sampling (Supplemental Table S6). In contrast, the relative proportion of IL-10 was significantly higher in primigravid women in both cohorts (p = 0.0033 (Fig. 3b), [Mann Whitney U test]) and in the PROMOTE cohort (p = 0.0193 [Mann Whitney U test]) but not in DPSP (Supplemental Figure S10) nor after multivariable adjustment (Supplemental Table S6). The relative proportion of IFNγ expressing nnCD4^+^ was similar between primigravid and multigravid women in both cohorts. In contrast to the malaria-specific nnCD4^+^ T cell compartment, only a low percentage of malaria-specific CD8^+^ T cells expressed cytokines and did not significantly differ across gravidity (Supplemental Figure S9c). Together, these data suggest that both the overall magnitude, and relative proportion, of malaria-specific nnCD4^+^ T cell responses differ across gravidity, with primigravid women tending to have a more IL-10 producing response, and multigravid women tending to have a more TNFα producing response.

Among PROMOTE women, a second blood sample was obtained at the time of delivery, offering us the opportunity to compare the impact of different IPTp regimens (IPTp-SP vs. IPTp-DP) on malaria-specific T cell responses at delivery. Interestingly, there was no difference in malaria-specific nnCD4^+^ T cell responses between IPTp groups at the time of enrolment or delivery (Supplemental Figure S11). Furthermore, nnCD4^+^ T cell responses between enrolment and delivery amongst primigravid and multigravid women were similar (Supplemental Figure S12).

We next evaluated whether percentages of malaria-specific nnCD4^+^ T cell populations correlated with clinical outcomes during pregnancy or at delivery. This analysis was restricted to PROMOTE women receiving IPTp-SP, since IPTp-DP significantly reduced parasite prevalence in the PROMOTE cohort,^62^ and the DPSP cohort is ongoing and remains blinded. We observed a significant positive correlation between the relative proportion of malaria-specific nnCD4^+^ T cells producing IL-10 and *Pf* parasite prevalence during pregnancy (R_s_ = 0.55, p = 0.00014, Fig. 3c [spearman correlation]), and this association remained significant after adjusting for gravidity, maternal age, and parasitaemia status at the time of enrolment (Supplemental Table S7). In contrast we observed a negative correlation between the relative proportion of malaria-specific nnCD4^+^ T cells producing TNFα and parasite prevalence (R_s_ = −0.35, p = 0.023 [spearman correlation]), although this did not remain not significant on multivariable analysis (Supplemental Table S7). No significant correlation was observed between the relative proportion of malaria-specific nnCD4^+^ T cells producing IFNγ and parasite prevalence during pregnancy (R_s_ = −0.15, p = 0.35, Fig. 3c, Supplemental Table S7 [spearman correlation]). Similar associations were observed when considering the overall magnitude (Supplemental Table S8). Interestingly, both the percentage of IL-10^+^ nnCD4^+^ T cells (p = 0.0069, Fig. 3d [Mann Whitney U test]) and the relative proportion of nnCD4^+^ T cells producing IL-10 (p = 0.0028, Fig. 3e [Mann Whitney U test]) were higher in women who had PM (defined by placental blood being LAMP positive for *Pf*). Associations between the relative proportion of IL-10 producing cells and PM remained significant after adjusting for gravidity, maternal age, and parasitaemia status at the time of enrolment (Supplemental Table S9), although associations between the percentage of IL-10^+^ nnCD4^+^ T cells and PM did not (Supplementary Table S10). Similar trends were seen when comparing women with and without histopathologic evidence of PM (Supplemental Figure S13a and b).

To determine whether the identified gravidity-dependent differences were specific to malaria, we took advantage of a unique opportunity in the DPSP cohort, in which a significant proportion of women were seropositive for SARS-CoV-2 at enrolment. We collected PBMCs from women who tested seropositive for SARS-CoV-2 and stimulated with PepTivator SARS-CoV-2 peptides or phorbol myristate acetate (PMA) (positive control) and assessed for intracellular production of IFNγ, TNFɑ, and IL-10. There were no gravidity-dependent differences in cytokine production to SARS-CoV-2 S protein (Supplemental Figure S14a and b). Primigravid women had higher IL-10 producing nnCD4^+^ T cells following stimulation with PMA, but this may have reflected recent malaria-induced activation (Supplemental Figure S14c and d).^26^

Together, these results suggest that primigravid women have a malaria-specific CD4^+^ T cell response that is skewed towards IL-10 and is associated with a higher risk of malaria in pregnancy. In contrast, multigravid women have a response skewed towards TNFα, and this response may correlate with protection against malaria in pregnancy.

### Malaria-specific IFNγ/IL-10 co-producing nnCD4^+^ T cells are more frequently observed in primigravid women

**Fig. 4:**
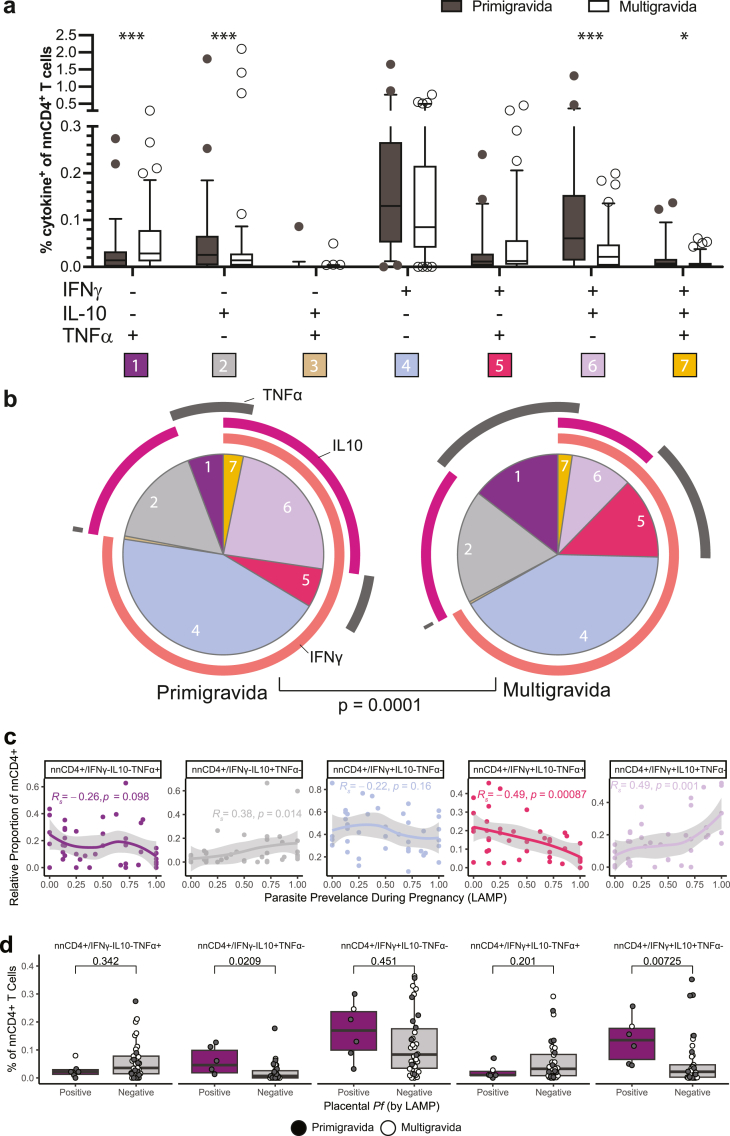
**Identification of distinct malaria-specific CD4**^**+**^**T cell subsets that differ across gravidity**. (a) The percentage of each individual combination of TNFα, IFNγ and IL-10 producing nnCD4^+^ T cells of individuals from DPSP and PROMOTE combined (primigravid n = 49, grey, multigravid n = 85, white). (b) The pie charts show the relative proportion (mean) of each individual combination of cytokine-producing nnCD4^+^ T cells among the total population of malaria-specific cells. The p-value shown under the pie chart is calculated using a partial permutation test. (c) Correlations between relative proportion of malaria-specific, cytokine^+^ nnCD4^+^ T cells (n = 43) of the top 5 largest subsets with *Pf* parasite prevalence during pregnancy detected by LAMP among women receiving SP chemoprevention (n = 43, PROMOTE). Locally estimated scatterplot smoothing (LOESS) were fit to the relative proportion of nnCD4^+^ expression. The p-values and R-values were calculated using Spearman correlations. (d) The percentage of malaria-specific nnCD4^+^ subsets producing combinations of IFNγ, IL-10, and TNFα stratified by placental malaria status as measured by placental blood LAMP (LAMP positive n = 6, LAMP negative n = 37). Data shown are background (Media) subtracted. p-values are calculated using a Mann Whitney U test. ∗ = p < 0.05, ∗∗ = p < 0.01, ∗∗∗ = p < 0.001.

To further characterise nnCD4^+^ T cell subsets, we performed Boolean gating to identify the percentage of cells expressing each combination of cytokines. In both cohorts, primigravid women had significantly higher percentages of malaria-specific nnCD4^+^ T cells co-producing IFNγ and IL-10 (IFNγ^+^IL-10^+^TNFα^−^) compared to multigravid women, even after adjusting for maternal age and parasitaemia at enrolment (p = 0.000023, Fig. 4a; Supplemental Figure S15; Supplemental Table S5 [Mann Whitney U test]). Conversely, multigravid women had significantly higher percentages of malaria-specific nnCD4^+^ T cells producing TNFα alone (IFNγ^−^IL-10^−^TNFα^+^) (p = 0.00052, Fig. 4a; Supplemental Figure S15; Supplemental Table S5 [Mann Whitney U test]). When comparing the proportion of each cytokine combination amongst the total malaria-specific nnCD4^+^ T cell population, the trend was similar. Among primigravid women, IL-10 producing nnCD4^+^ T cells (including IL-10 single-producers and IFNγ^+^IL-10^+^ double producers) comprised a greater proportion of the malaria-specific response whereas among multigravid women, TNFα-producing nnCD4^+^ T cells (including TNFα single-producers and TNFα^+^ IFNγ^+^ double producers) comprised the greater proportion (p = 0.0001, Fig. 4b, Supplemental Table S6 [Mann Whitney U test]).

When assessing associations between malaria-specific nnCD4^+^ T cell subsets and clinical outcomes during pregnancy among PROMOTE women given IPTp-SP, results were consistent with the individual cytokine analysis. Higher relative proportion of IL-10^+^IFNγ^+^TNFα^−^ cells at enrolment correlated with higher parasite prevalence during pregnancy (R_s_ = 0.49, p = 0.001, Fig. 4c [spearman correlation]). Percentages of IL-10^+^IFNγ^+^TNFα^−^ cells were also significantly higher in placental blood that was LAMP positive for *Pf* at delivery (Fig. 4d). Similar trends were seen after multivariable adjustment (Supplemental Tables S9, S10) and when comparing women with and without histopathologic evidence of PM (Supplemental Figure S13c). These results suggest that malaria-specific IL-10^+^IFNγ^+^ co-producing nnCD4^+^ T cells are associated with a higher burden of malaria in pregnancy.

### IL-10^+^IFNγ^+^TNFα^−^ CD4^+^ T cells express a Tr1 gene signature

**Fig. 5:**
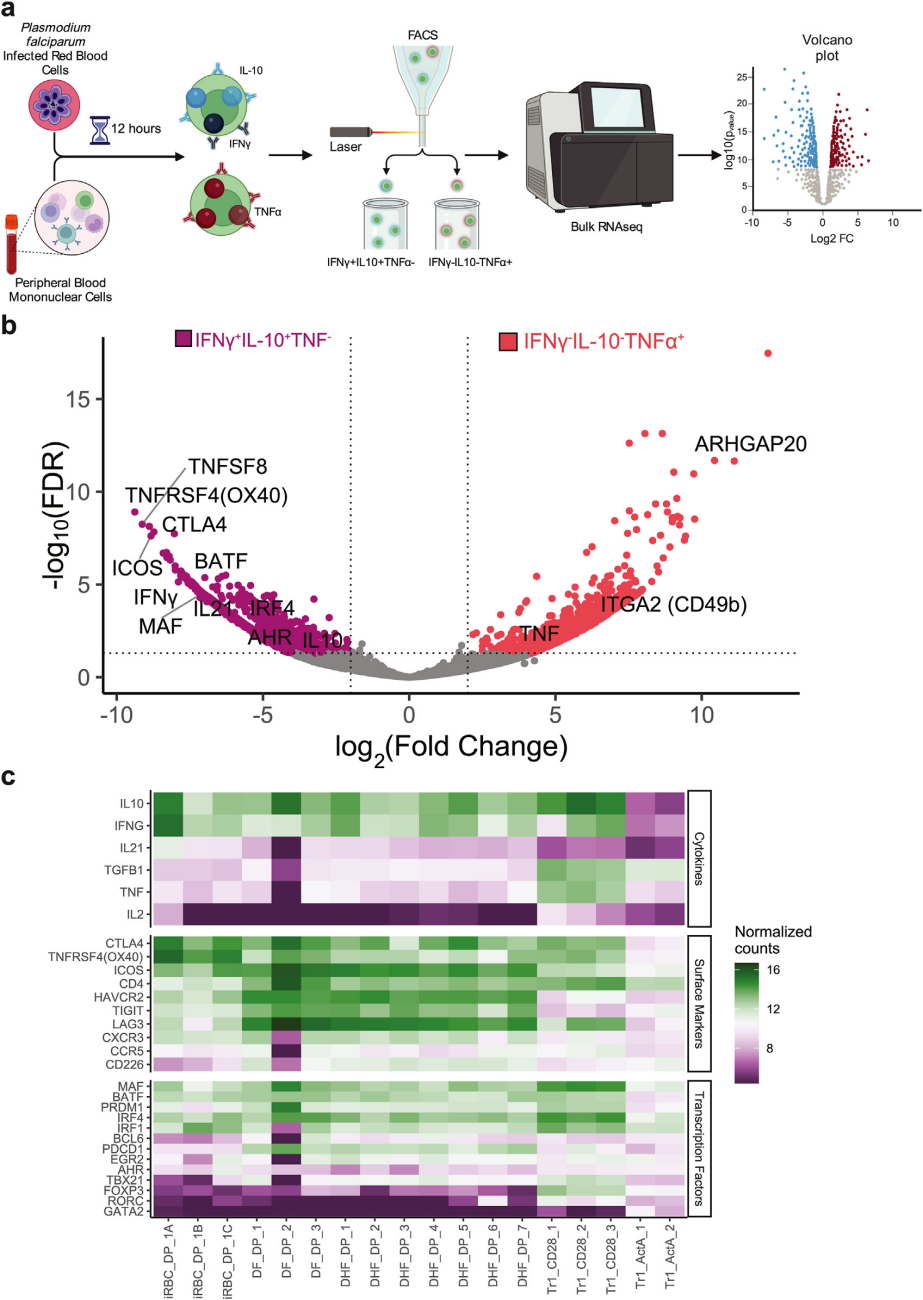
**DEGs associated with IL-10**^**+**^**IFNγ**^**+**^**nnCD4**^**+**^**cells and TNFα**^**+**^**nnCD4**^**+**^**cells**. (a) Schematic of cytokine capture experiment. PBMCs were obtained from 3 pregnant women in DPSP and sorted after a 12-h stimulation with iRBC. (b) Differentially expressed genes from bulk RNA sequencing from cells sorted for expression IL-10^+^IFNγ^+^TNFα^−^ (n = 3, purple) or IL-10^−^IFNγ^-^TNFα^+^ (n = 3, red) are depicted in a volcano plot. (c) Cytokines, surface markers, and transcription factors characteristic of Tr1 cells with normalized counts across Tr1 cells from public datasets with dengue fever (DP), dengue haemorrhagic fever (DHF), and T cells stimulated with Activin A (ActA) and CD3/CD28 beads (CD28).

To further characterise malaria-specific nnCD4^+^ T cell subsets at a molecular level, we performed RNA sequencing on IL-10^+^IFNγ^+^TNFα^−^ and IL-10^−^IFNγ^-^TNFα^+^ nnCD4^+^ T cells isolated from three DPSP primigravid pregnant women at enrolment using a cytokine capture assay (>200 cells per sample, Supplemental Table S11, Fig. 5a). Compared with TNFα^+^ cells, IFNγ^+^IL-10^+^ double positive cells had higher expression of 2408 DEGs (log 2(FC) > 2 (p < 0.1), Supplemental Table S12). Interestingly, the highest DEGs among IFNγ^+^IL-10^+^ double positive cells were *TNFRSF4* (*OX40*) and *ICOS*—both of which were identified as elevated in primigravid women in our CD4^+^CD45RA^−^ AIM assay. In addition, the expression patterns of IL-10^+^ and IFNγ^+^ nnCD4^+^ T cells recapitulated expression patterns of Tr1 cells. For instance, these cells expressed *CTLA4*, a peripheral tolerance co-receptor, and the putative transcription factors, *BATF* and *c-MAF*, both of which have been shown to be crucial to adaptive tolerance in the context of T cells (Fig. 5b and c).^63^ These cells also expressed the cytokine *IL-21*, which, although conventionally thought to be involved in Tfh cell development and B cell help, has also been shown to be expressed by Tr1 cells.^64^ We confirmed that malaria-specific IL-10/IFNγ co-producing cells also produce IL-21 using a flow cytometric assay (Supplemental Figure S16).

We further wanted to confirm that specific IL-10/IFNγ co-producing cells are indeed Tr1 cells. To do so, we compared gene expression patterns with recently published datasets evaluating Tr1 cells in a few different contexts (Dengue fever, Dengue haemorrhagic fever, and activation with CD3/CD28 beads and Activin A). In this meta-analysis, we found high expression of known Tr1 cytokines (*IL-10, IFNγ*), surface receptors (*CTLA4*, *LAG3*, and *ICOS*), and transcription factors (*cMAF, BATF, PRDM1*). The double positive IFNγ^+^IL-10^+^ population matched published expression patterns of Activin A and CD3/CD28 costimulated beads (Fig. 5c). Together, these results indicate that the positive IFNγ^+^IL-10^+^ population are indeed Tr1 cells.

TNFα single positive cells showed upregulation of 979 DEGs in comparison to the IFNγ^+^IL-10^+^ cell population (Fig. 5b, Supplemental Table S12). Along with higher expression of *TNFα*, these cells also expressed *ITGA2* (CD49b), a transmembrane receptor to collagen and *ARHGAP20* a rho GTPase activator consistent with a bias towards peripheral Treg differentiation.^65^ While the importance of these TNFα expressing nnCD4^+^ T cells remains unclear, their presence in multigravid populations suggests a role in gravidity-dependent acquired immunity against malaria.

## Discussion

In this study of pregnancy-specific malarial immunity in a high transmission setting in eastern Uganda, we identified a shift in the quality of the malaria-specific nnCD4^+^ T cell response with increasing gravidity. Primigravid women had higher percentages of malaria-specific, nnCD4^+^ T cells that co-express IL-10 and IFNγ compared with multigravid women. Higher percentages of these cells were associated with parasitaemia during pregnancy and PM. These IL-10 and IFNγ co-producing nnCD4^+^ T cells had genomic features suggestive of Tr1 cells, including expression of the transcription factors *cMAF* and *BATF* and the cell surface makers *CTLA4* and *LAG-3*. In contrast, multigravid women had higher percentages of CD4^+^ T cells that produced TNFα in the absence of IFNγ, and these cells were associated with protection against malaria in pregnancy.

We initially used an unbiased, whole blood RNA-sequencing approach to compare the cellular immune response to malaria between primigravid and multigravid women. Though we observed significant differences in gene expression between pregnant and non-pregnant Ugandan women, consistent with published reports,^55^^,^^56^ we did not observe significant differences in gene expression between primigravid and multigravid women at a bulk level. However, when only considering *Pf* parasitemic women at enrolment to restrict our analysis to women with known exposure to malaria in pregnancy, we found differential upregulation of several genes, including *TSLP*. *TSLP* has been reported to be expressed by placental cytotrophoblasts in early placentation and to induce regulatory CD4^+^ T cells by activating dendritic cells.^66^ Outside of pregnancy, expression of *TSLP* in PBMCs was higher in asymptomatic patients with malaria compared to severe cases.^66^ We hypothesize that, in first pregnancies in *Pf* -exposed women, TSLP is produced by shed placental cytotrophoblasts and plays a role in T cell differentiation; yet this remains to be determined.

We next focused our analysis on the role of CD4^+^ T cells in gravidity-dependent malarial immunity, given their critical role in coordinating responses that regulate or galvanise the immune response to antigens.^14^^,^^67^^,^^68^ Although percentages of circulating memory CD4^+^ T cells, FoxP3^+^ Tregs, and Tfh did not differ between primigravid and multigravid women, we found several notable differences in the malaria-specific response between groups. In particular, we identified higher percentages of activated malaria-specific CD4^+^ T cells expressing OX40, CD137, and ICOS in primigravid compared with multigravid women. Notably, OX40 signalling has been shown to play an important role in T cell differentiation and parasite control following *Plasmodium* infection in mice by promoting both helper T cell differentiation and anti-humoral *Plasmodium* immunity.^69^ We did not observe any significant correlations between percentages of OX40^+^ malaria-specific CD4^+^ T cells and VAR2CSA-specific antibody responses measured concurrently, similar to results of another study.^32^

In two independent cohorts of pregnant women, we found that primigravid women had a higher proportion of IFNγ^+^IL-10^+^TNFα^−^ cells compared with multigravid women. These differences were malaria-specific, as responses to SARS-CoV-2 peptides among COVID-19 exposed participants did not significantly differ between primigravid and multigravid women. Using transcriptional profiling, we further found that these cells expressed surface markers *CTLA4, OX40, ICOS*, and *LAG3* in combination with the transcription factors *BATF* and *MAF* but not *FOXP3,* altogether consistent with a Tr1 signature that recently was conserved across multiple infection models and species.^25^ Tr1 cells are thought to play a critical role in tempering the immune response to pathogens such as *Plasmodium* by suppressing the inflammatory programmes of myeloid cells and other T cells.^70^^,^^71^ Indeed, mice with a T cell-targeted IL-10 deficiency are susceptible to greater disease severity following *Plasmodium* infection,^20^ and we previously reported that nnCD4^+^ production of IL-10 was associated with decreased disease severity in malaria-exposed children.^72^ A previous study described an increase in IFNγ/IL-10 co-producing nnCD4^+^ T cells in pregnant versus non-pregnant women living in Papua New Guinea, although differences between gravidities were not described.^32^ In that study, the authors observed a positive correlation between IL-10 producing T cells and haemoglobin levels at delivery, suggesting a protective role against disease. In our study, we speculate that higher frequencies of malaria-specific Tr1 cells in primigravid women may also prevent immune-related pathology among women newly exposed to sequestered VAR2CSA-expressing parasites, as most women in this study did not report symptoms despite parasitaemia.

Although Tr1 cells may play a role in protection against immunopathology, they may also facilitate parasite persistence by suppressing anti-parasite immunity, as has been shown in studies of leishmaniasis^73^, ^74^, ^75^ and toxoplasmosis.^21^^,^^76^ Consistent with this hypothesis, in our study, we observed that higher percentages of Tr1 cells correlated with higher prevalence of subsequent parasitaemia during pregnancy and higher risk of PM at delivery. In the Papua New Guinea study described above, the prevalence of *Plasmodium* infection was relatively low, limiting analyses between Tr1 cells and correlates of protection against malaria in pregnancy.^32^

Malaria-specific nnCD4 + T cells produced more TNFα in multigravid compared to primigravid women. Although TNFα has been proposed as a potential biomarker for severe malaria in non-pregnant individuals, it appears to correlate with protection in the context of pregnancy.^77^ These data are consistent with the hypothesis that TNFα reduces erythrocyte invasion,^78^ and malaria-specific nnCD4^+^ T cell production of TNFα has been correlated with protection following both natural infection^26^ and RTS,S vaccination.^79^, ^80^, ^81^, ^82^ Together, these data suggest that malaria-specific TNFα-producing nnCD4^+^ T cells may contribute to protection from malaria in pregnancy, though further research is needed to determine the factors involved in differentiation of these cells. Furthermore, understanding factors that limit Tr1 induction—and promote CD4+ T cell production of TNFα—may help in the rational design of effective vaccines in malaria-endemic settings.

There are several limitations to this study. First, not all assays were performed on every participant due to limited sample availability. However, we performed several assays across two independent cohorts, improving the generalisability of our findings. Second, most assays were performed utilising blood collected at enrolment during the second trimester, which may not have fully captured the immune response across pregnancy. However, among PROMOTE women, we were additionally able to evaluate cytokine responses at the time of delivery and found no significant differences either between IPTp arms nor between enrolment and delivery timepoints. Third, IPTp was administered to study participants, which may have independently influenced outcomes. However, our analysis of protection was only among PROMOTE women who received IPTp-SP, since DP significantly reduced parasite prevalence in the PROMOTE cohort.^62^ While the effects of IPTp-SP on the immunity of pregnant women is understudied, IPTp-SP has not been shown to modify immunity to *Pf* with respect to chemokines, intracellular cytokine responses, or antibody production.^83^^,^^84^ Fourth, we lacked data on the timing of parasite infection and PM establishment, but the timing of immune assessments was consistent in all women. Finally, the study focused solely on peripheral blood samples, which limits our ability to fully understand the role of CD4^+^ T cells in the placental compartment.^85^^,^^86^ Percentages of CD4^+^ T cells and CD45RO^−^ memory like T cells have been shown to be higher in peripheral than placental blood in malaria exposed pregnant women.^87^ There is also evidence that IL-10 production in placental tissue is higher in primigravid compared to multigravid women, and that peripheral IL-10 could be a biomarker of PM.^88^, ^89^, ^90^

Altogether, these findings suggest an important role for malaria-specific Tr1 cells in the immune response to malaria during first pregnancies, and that these cells may play a role in the pathogenesis of PM. Understanding whether suppression of Tr1 cells helps enable gravidity-dependent immunity against malaria in pregnancy may help facilitate the development of vaccines and other therapeutic interventions to protect against malaria in pregnancy.

## Contributors

PJ, ASK, MT conceived of the study. AK, JK, MM, FN, KM, and MK oversaw the clinical studies and/or collected/processed patient samples with input from GD and PJ. ASK, KvdP, MT, and LP coordinated the laboratory work. SD and BG provided VAR2CSA reagent and assisted with VAR2CSA ELISA protocols. ASK, KvdP, AD, MT, LP, KP, SB, conducted the experiments and data analysis with input from PJ. ASK and PJ drafted the manuscript with feedback from all authors including PR and MF. ASK, AD, SB, and PJ have verified the underlying data. All authors have read and support the findings presented in the manuscript.

## Data sharing statement

The data that supports the genomics findings in this study have been deposited in Gene Expression Omnibus GSE234585 (Whole Blood RNA-seq) and GSE234586 (PBMCs RNA-seq).

Upon reasonable request, deidentified participant data, experimental data, and data dictionary can be shared with other researchers. Data will be made available after approval of a study proposal and after signing a data access agreement. Please contact the corresponding author for more information.

## Declaration of interests

All authors declare no competing interests.
