# Supplementary material for: Malaria-specific Type 1 regulatory T cells are more abundant in first pregnancies and associated with placental malaria
Source: eBioMedicine. 2023 Aug 25;95:104772. doi: 10.1016/j.ebiom.2023.104772 (PMC10474374; doi:10.1016/j.ebiom.2023.104772)
Supplement: Supplemental Figures and Tables [file mmc1.docx]

Supplemental Materials

Supplemental Figure 1: Directed acyclic graph of factors influencing Malaria-specific T cells and pregnancy outcome 3

Supplemental Figure 2: VAR2CSA levels of primigravid and multigravid women by parasitaemia status. 4

Supplemental Figure 3: Whole blood RNA sequencing comparing qPCR+ and qPCR- women. 5

Supplemental Figure 4: Gating strategy for flow cytometry analysis of peripheral blood mononuclear cells6

Supplemental Figure 5: Flow cytometry characterization of T cell subsets 7

Supplemental Figure 6: Activation induced marker assay comparison by malaria at enrolment. 8

Supplemental Figure 7: Age and gravidity explain differences in nnCD4^+^ T cells but age does not significantly correlate with activated nnCD4^+^ T Cells. 9

Supplemental Figure 8: Malaria-specific Tfh cells populations and associations between malaria-specific CD4^+^ T cell subsets and VAR2CSA-specific antibodies. 10

Supplemental Figure 9: Malaria-specific CD8+ T cell populations. 11

Supplemental Figure 10: Percentage and Relative Proportion of nnCD4^+^ T cells expressing cytokines in the DPSP and PROMOTE cohort. 12

Supplemental Figure 11: Comparison of cytokine producing CD4^+^ T cells at enrolment and delivery between IPTp arms among women in the PROMOTE clinical trial.. 13

Supplemental Figure 12: Comparison of T cell cytokine production at enrolment and delivery among primigravid and multigravid women. 14

Supplemental Figure 13: Comparison of nnCD4^+^ T cell subsets by placental malaria detected by histopathology. 15

Supplemental Figure 14: nnCD4+ T cell response to SARS-CoV-2 Spike peptide and phorbol myristate acetate (PMA).. 16

Supplemental Figure 15: Comparison of nnCD4^+^ T cell cytokine producing subset between primigravid and multigravid women in DPSP and PROMOTE separately. 17

Supplemental Figure 16: nnCD4+ T cells subsets including IL-21. 18-19

Supplemental Table 1: Whole blood RNAseq quality control metrics 20

Supplemental Table 2: List of genes differentially expressed in whole blood RNAseq among pregnant and non-pregnant women 20

Supplemental Table 3: Pathways upregulated in pregnant women 20

Supplemental Table 4: Differentially expressed genes in primigravida qPCR^+^ 20

Supplementary Table 5: Malaria-specific CD4^+^ T cell magnitude at enrolment and associations with gravidity 21

Supplementary Table 6: Malaria-specific CD4^+^ T cell proportions at enrolment and associations with gravidity. 22

Supplementary Table 7: Malaria-specific CD4^+^ T cell proportions at enrolment and associations with *Pf* parasite prevalence in pregnancy 23

Supplementary Table 8: Malaria-specific CD4^+^ T cell magnitude at enrolment and associations with *Pf* parasite prevalence in pregnancy 24

Supplementary Table 9: Malaria-specific CD4^+^ T cell proportions at enrolment and associations with placental malaria measured by *Plasmodium falciparum* detection by Loop-mediated isothermal amplification (LAMP) at delivery 25

Supplementary Table 10: Malaria-specific CD4^+^ T cell magnitude at enrolment and associations with placental malaria measured by *Plasmodium falciparum* detection by Loop-mediated isothermal amplification (LAMP) at delivery 26

Supplemental Table 11: Cytokine capture Cell counts 27

Supplemental Table 12: Differentially expressed genes from sorted cells 27

**
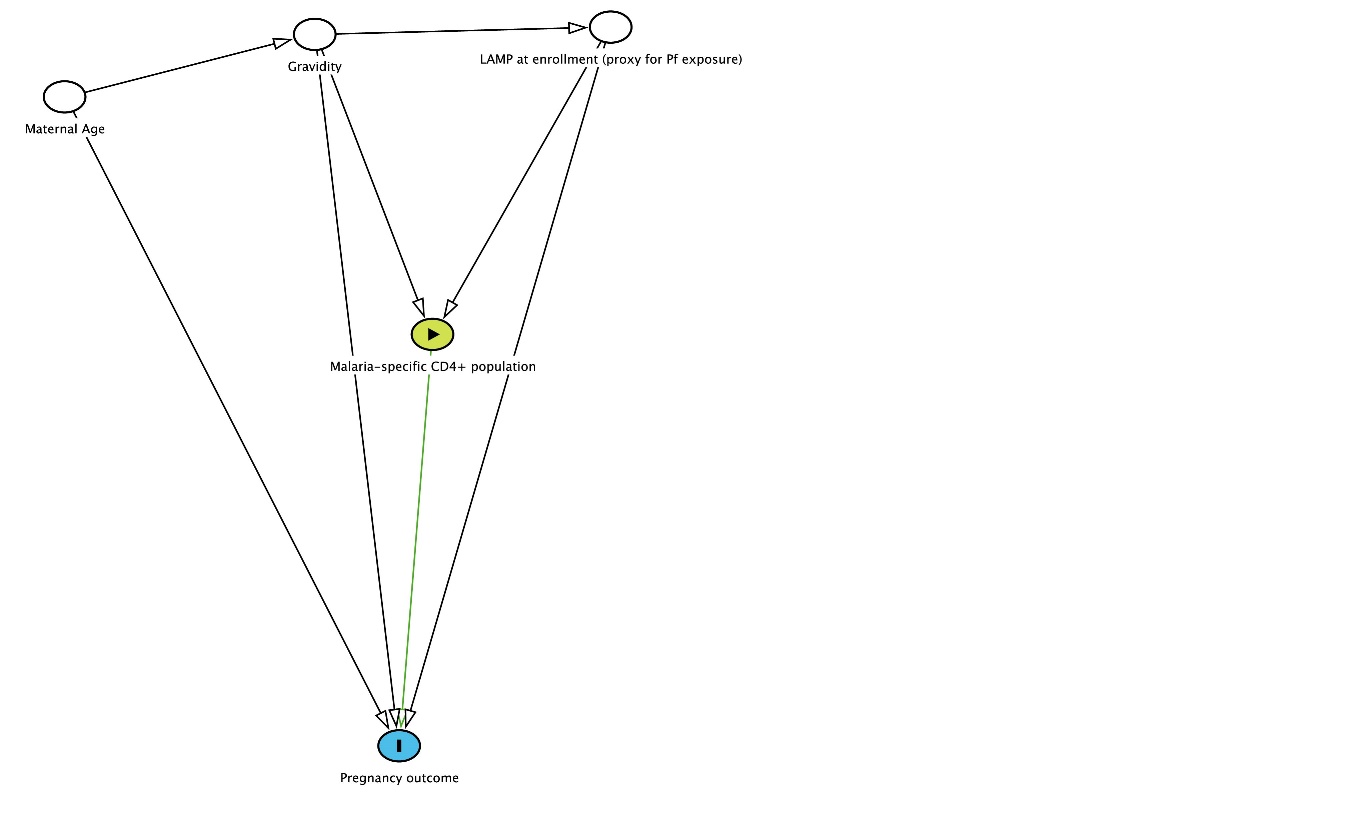
**

**Supplemental Figure 1: Directed acyclic graph of factors influencing Malaria-specific T cells and pregnancy outcome.**


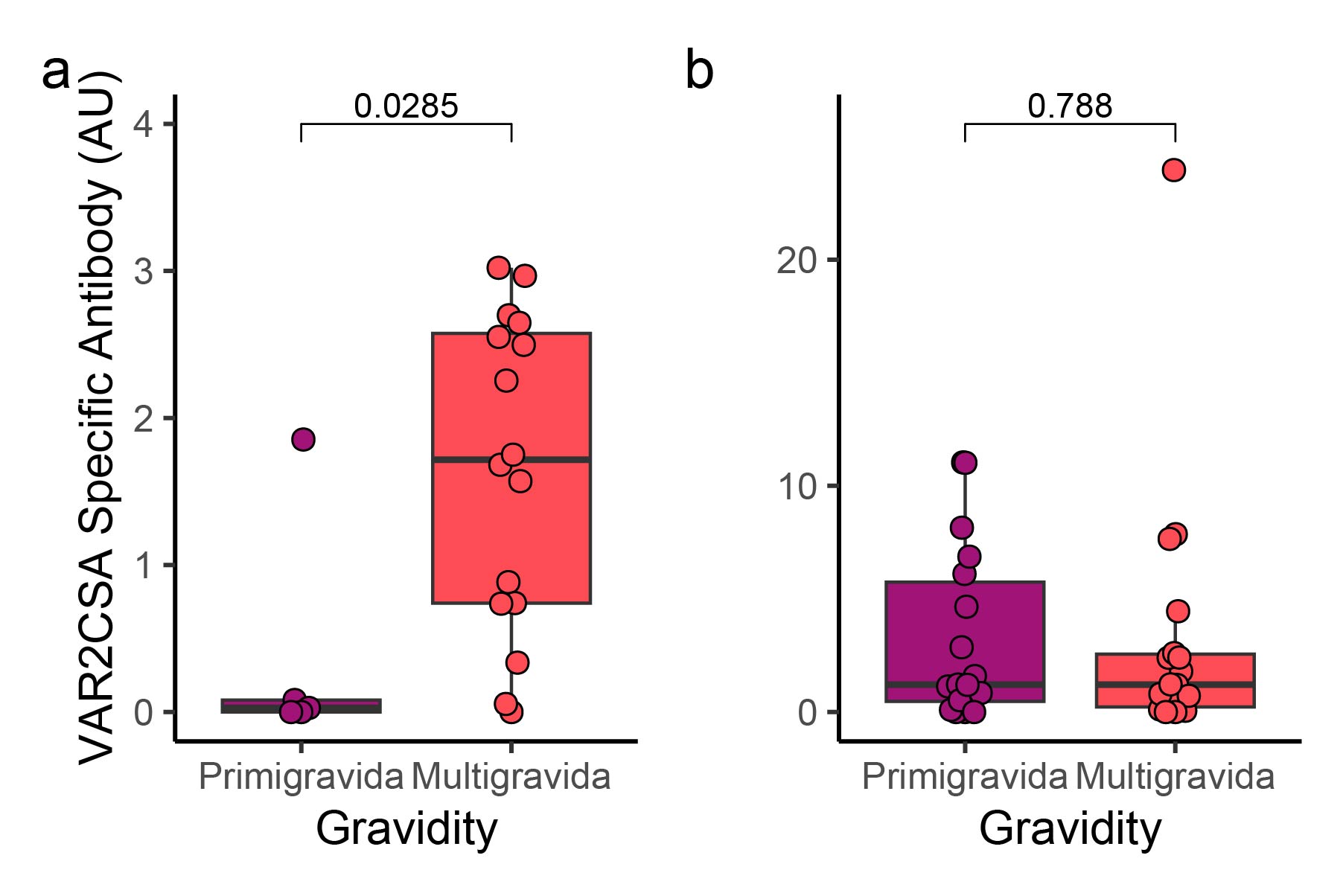


## **Supplemental Figure 2: VAR2CSA levels of primigravid and multigravid women by parasitaemia status. (a)** VAR2CSA levels of parasite negative mothers (by LAMP/qPCR) at enrolment (Primigravida, purple n = 5; Multigravida, red n = 16). (b) VAR2CSA levels of parasite positive mothers (by LAMP/qPCR) at enrolment (Primigravida, purple n = 18; Multigravida, red n = 18). P-values shown are from Mann Whitney U test.


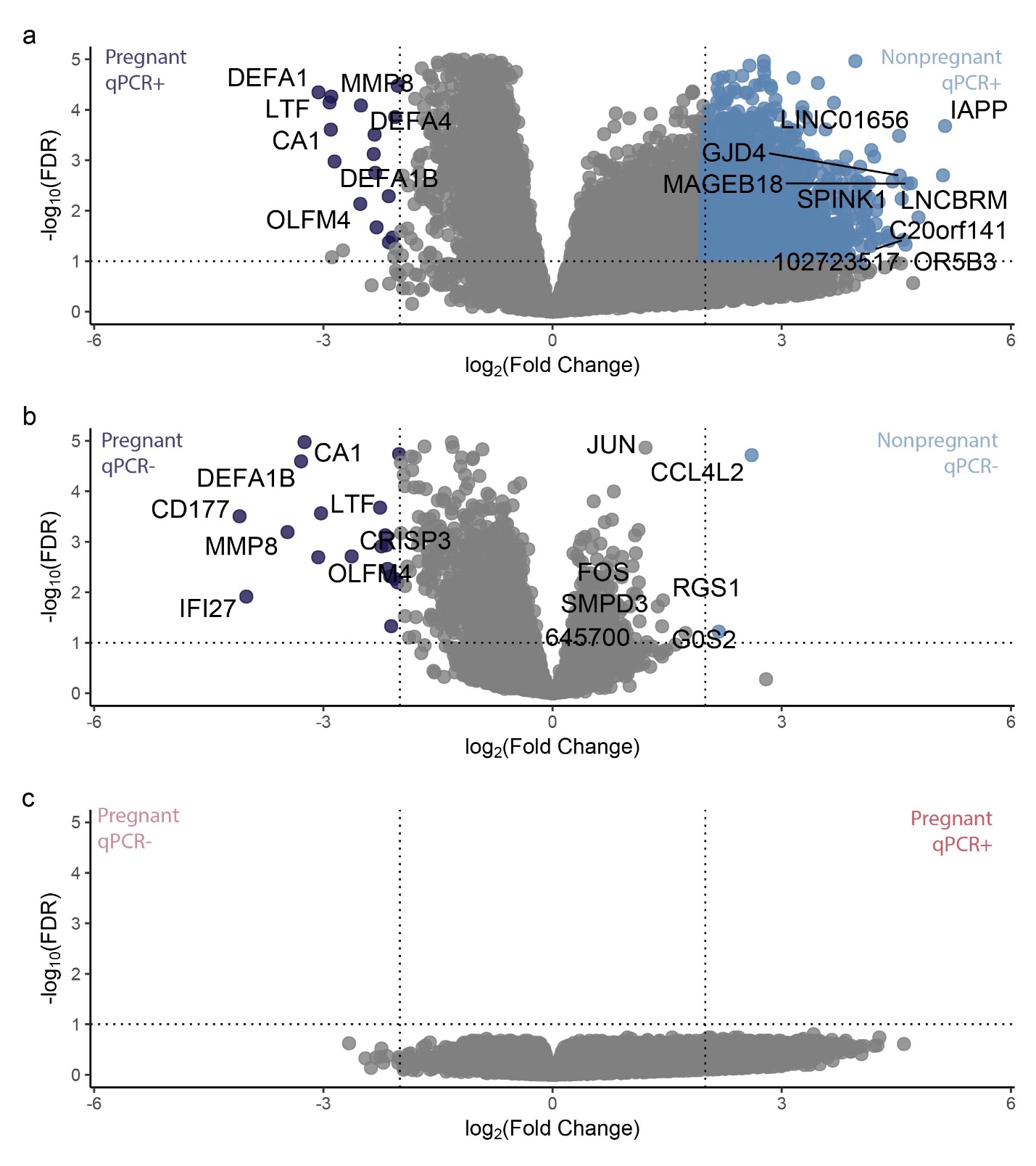


## **Supplemental Figure 3: Whole blood RNA sequencing comparing qPCR+ and qPCR- women.** Shown are volcano plots comparing (a) qPCR+ nonpregnant (n=5) and qPCR+ pregnant (n=13) (b) qPCR- pregnant (n=10) qPCR- nonpregnant (n=5) (c) qPCR+ pregnant (n=13) and qPCR- pregnant(n=10). Differentially expressed genes had an adjusted p-value (Benjamini Hochberg) < 0.1 and minimum log 2-fold change of 2. Selected genes were annotated in volcano plots.


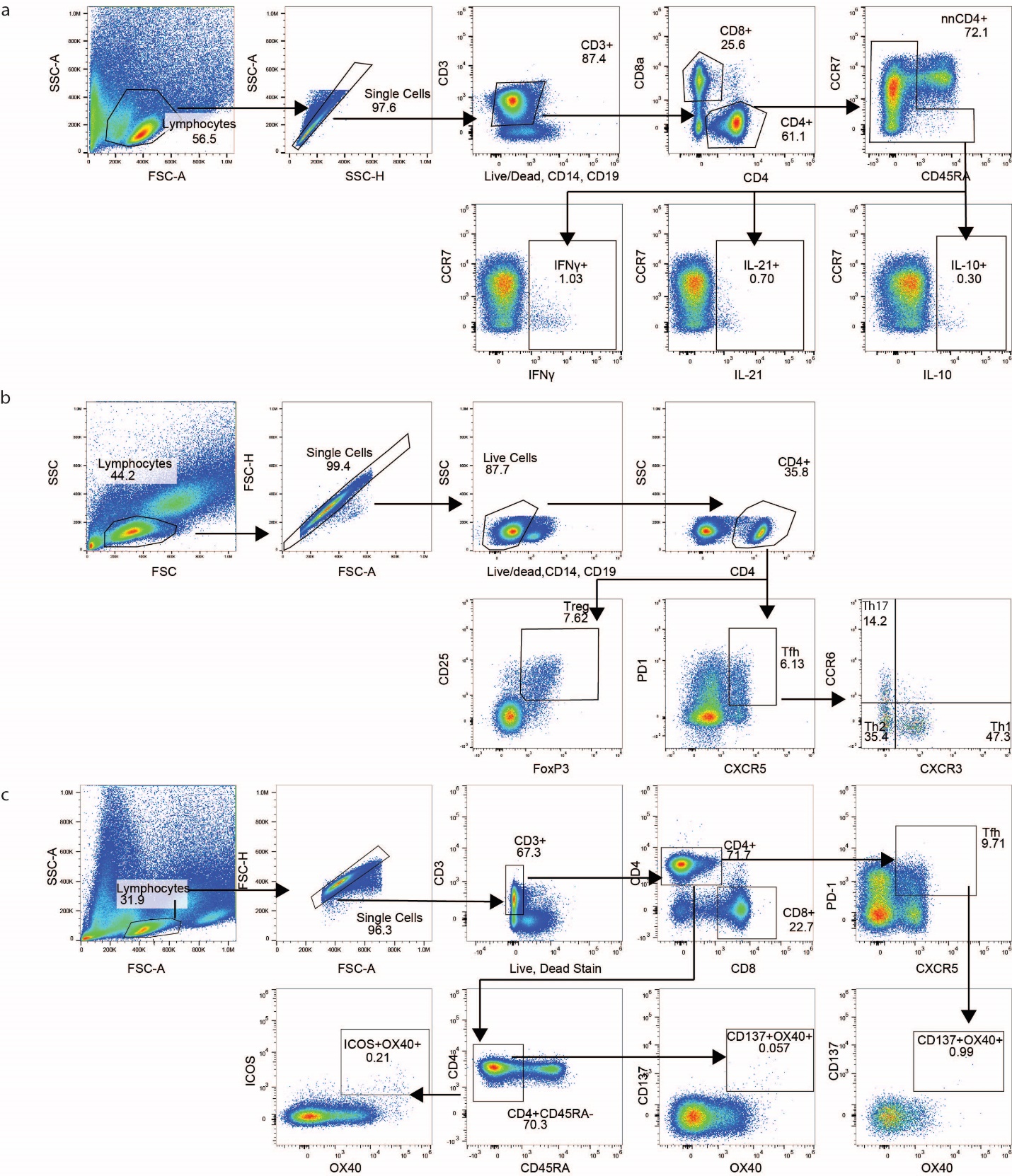


## Supplemental Figure 4: Gating strategy for flow cytometry analysis of peripheral blood mononuclear cells. Shown are representative dot plots of (a) intracellular cytokines staining, (b) unstimulated T cell subsets, and (c) activation induced markers assays.

**
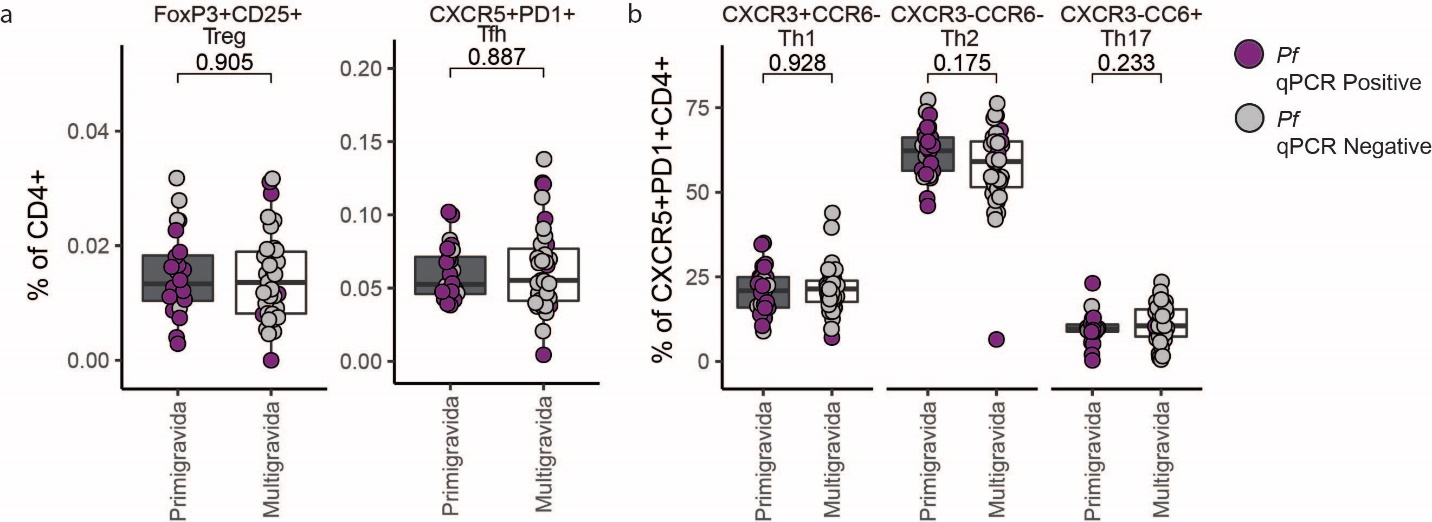
**

## Supplemental Figure 5: Flow cytometry characterization of T cell subsets

(a) Depicted are percentages of Treg and Tfh cells gated from CD4^+^ T cells, and (b) Th1, Th2 and Th17 T cell subsets gated from CXCR5^+^PD1^+^CD4^+^ T cells of primigravid (n=33) and multigravid (n=66) women. P-values are calculated by Mann Whitney U test.


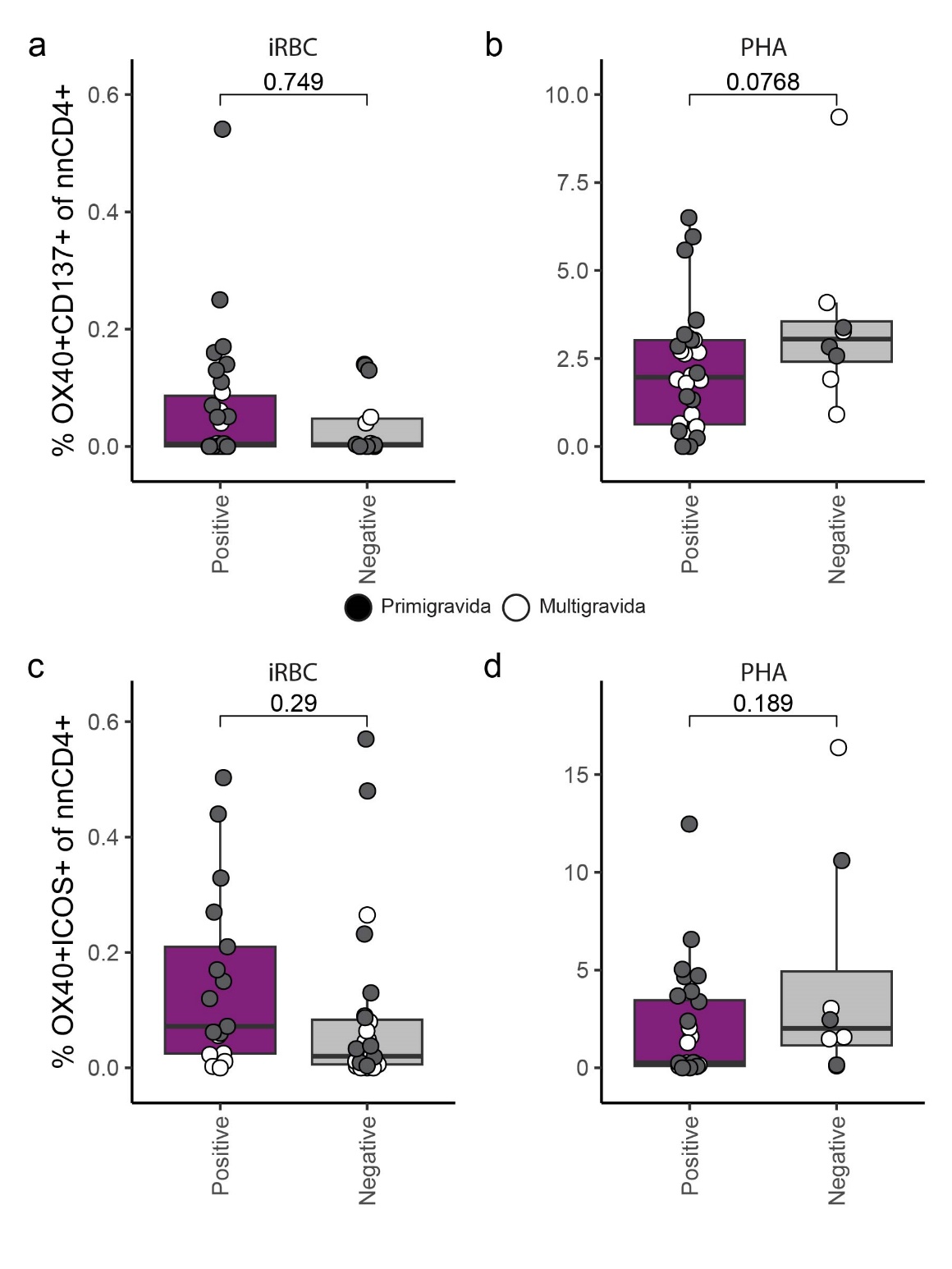


## Supplemental Figure 6: Activation induced marker assay comparison by malaria at enrolment. Percentage of CD45RA- CD4+ T cells expressing OX40 and (b) CD137 or (d) ICOS of *Pf* qPCR positive (purple, iRBC (n = 30) , PHA (n = 28)) or *Pf* qPCR negative (light grey, iRBC (n = 14) , PHA (n= 8)) stimulated by iRBC (left) and PHA (right) (dark grey= primigravida, white= multigravida) are depicted. P-values are calculated by Mann Whitney U test.


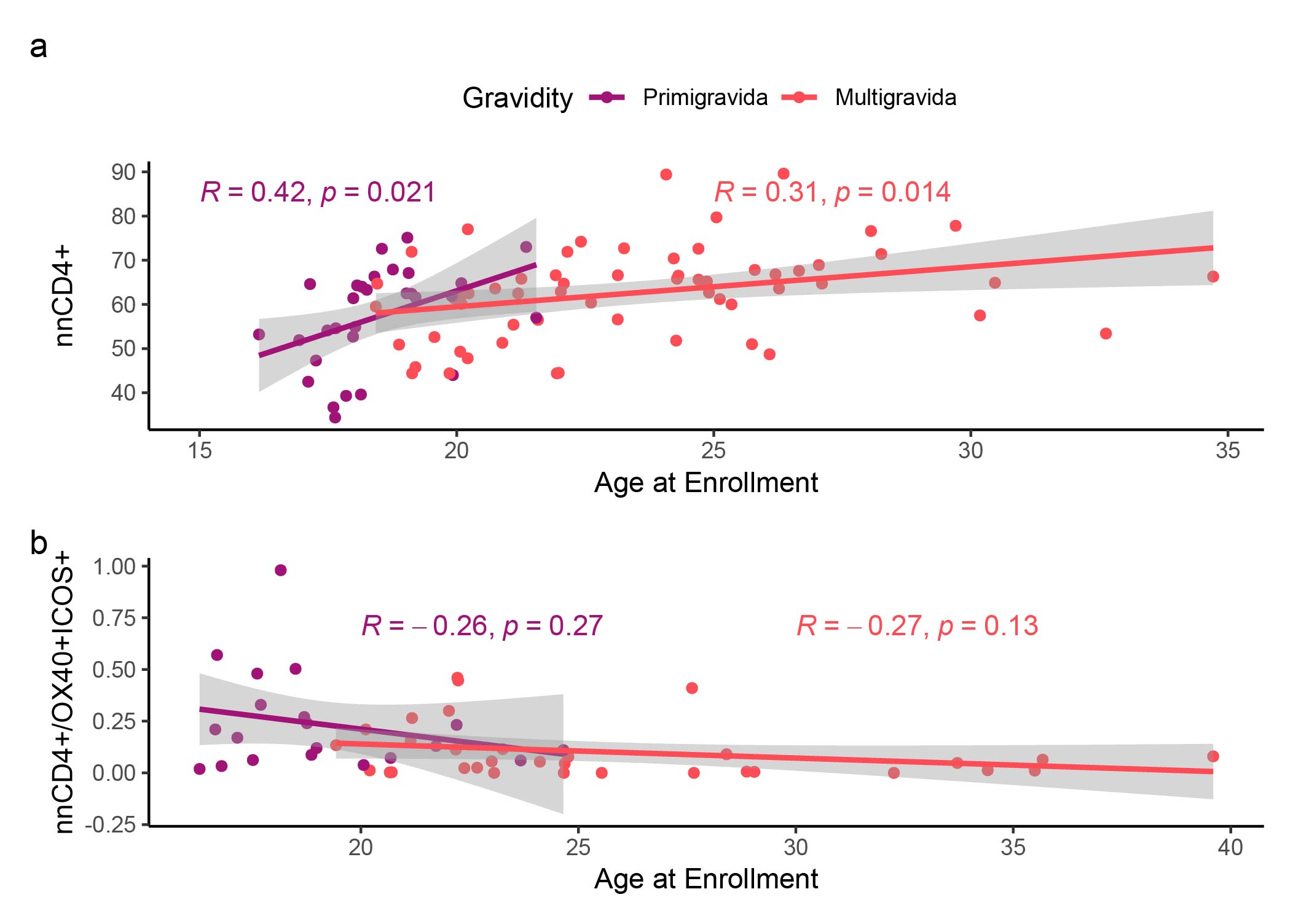


## Supplemental Figure 7: Age and gravidity explain differences in nnCD4^+^ T cells but age does not significantly correlate with activated nnCD4^+^ T Cells. Linear regression of age at enrolment and (a) nnCD4^+^ T cells in primigravid (n=62) and multigravid (n=98) women and (b) nnCD4^+^ T cells expressing OX40 and ICOS in response to iRBC in primigravid (n= 26) and multigravid (n=42) PBMCs are shown. R and p values depicted are from Pearson correlations.


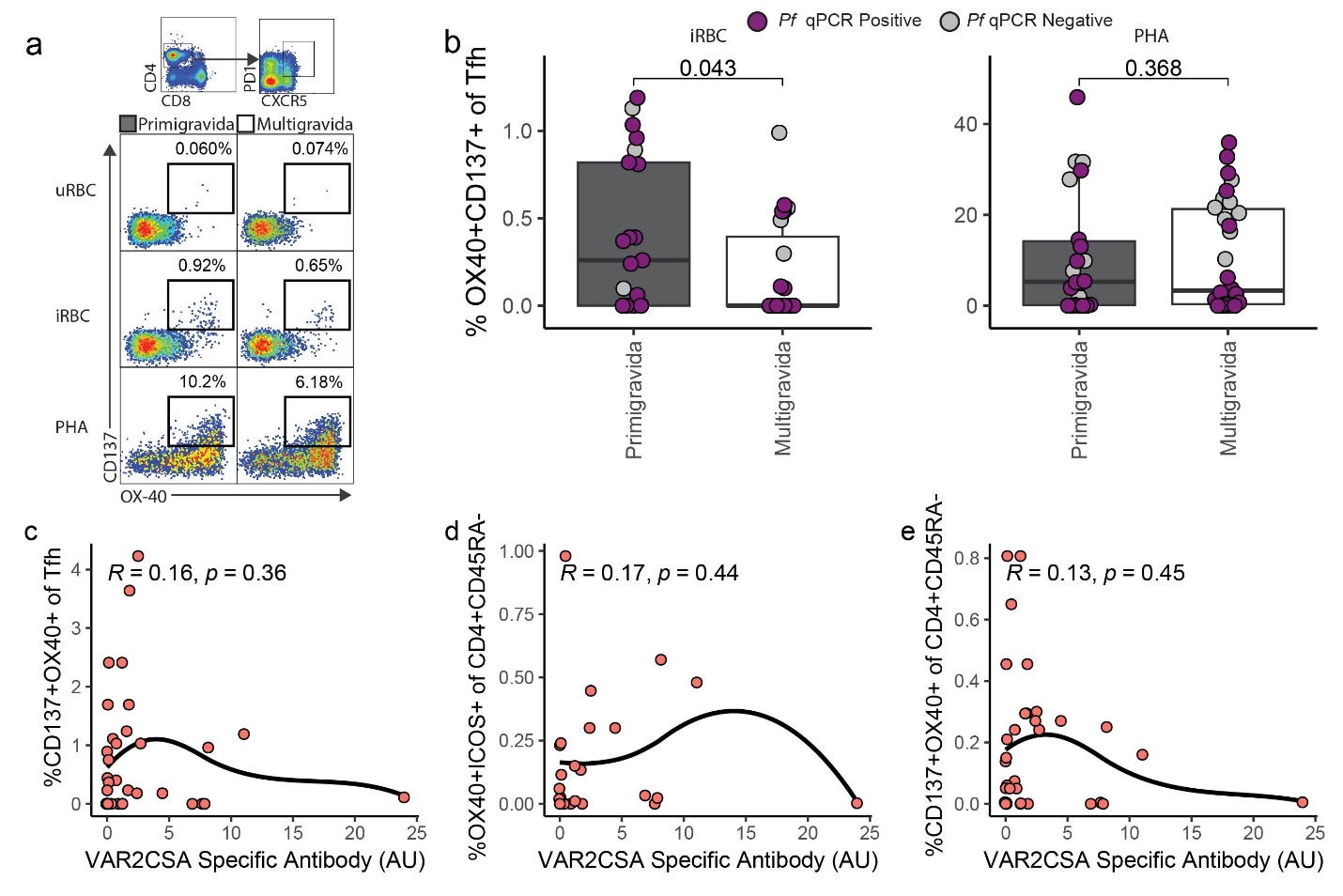


## Supplemental Figure 8: Malaria-specific Tfh cells populations and associations between malaria-specific CD4^+^ T cell subsets and VAR2CSA-specific antibodies. Shown are (a) dot plots of CD137^+^ OX40^+^ Tfh cells in response to uRBCs, iRBCs, and PHA analysed by flow cytometry. (b) Percentage of CD137^+^OX40^+^ Tfh cells by gravidity (primigravid, n=21 iRBC, n=20, multigravid, white, n=21 iRBC, n=17 PHA) are depicted. P-values are calculated by Mann Whitney U test. Spearman correlation between (c) Tfh/CD137^+^OX40^+^ (d) CD4^+^CD45RA^-^/ OX40^+^ICOS^+^ (n=26) (e) CD4^+^CD45RA^-^/CD137^+^OX40^+^ (n=30) stimulated with iRBC and VAR2CSA specific antibody titres at enrolment from the DPSP cohort.

**
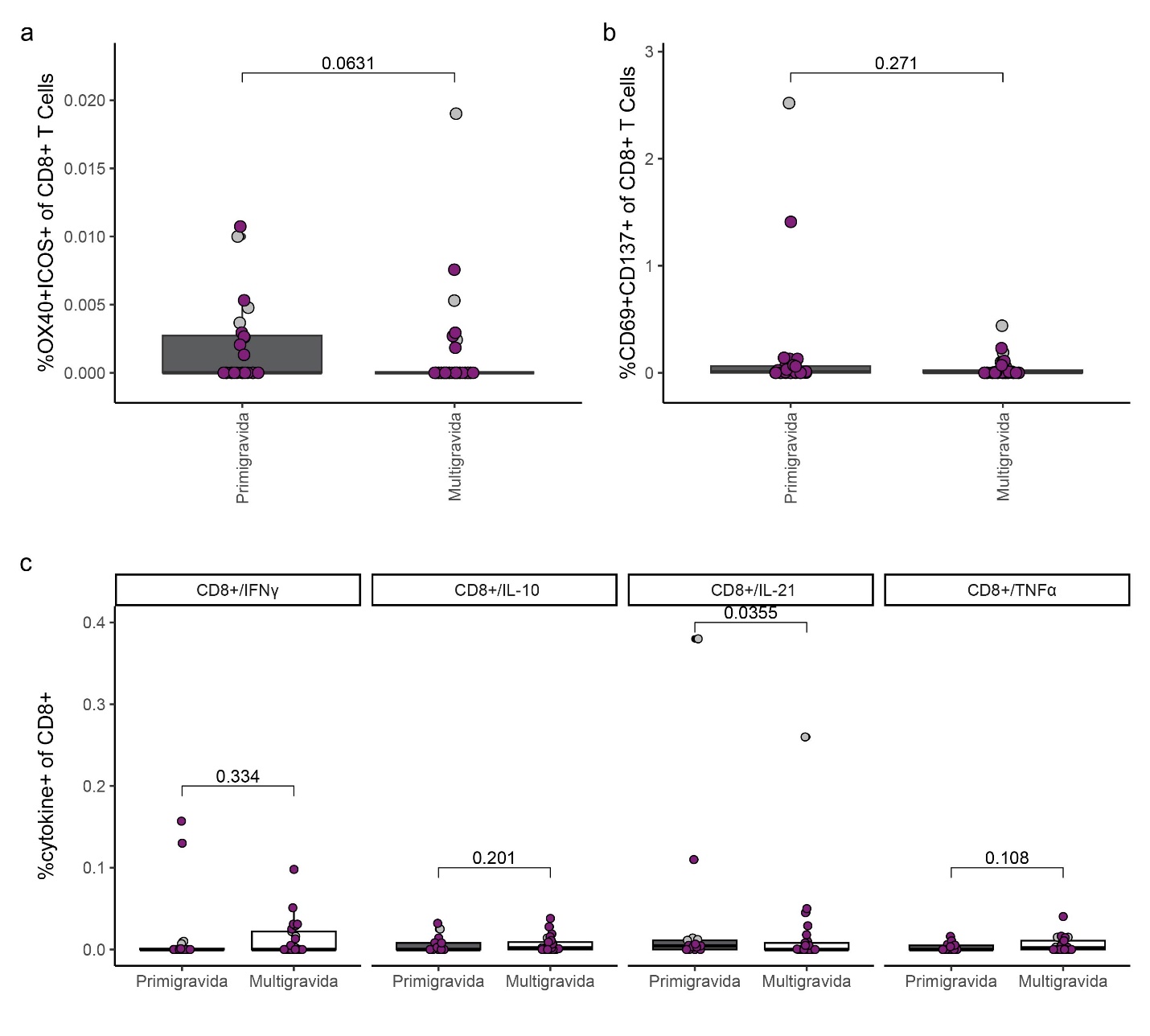
**

## Supplemental Figure 9: Malaria-specific CD8+ T cell populations. (a) CD8+ T cells stimulated with iRBC expressing ICOS and OX40 (primigravid, dark grey, n=19, multigravid, white, n=19) and (b) percentage of CD8+ T cells expressing indicated cytokines when stimulated with iRBC (primigravid, dark grey, n=16, multigravid, white, n=28) are shown. Analysed by flow cytometry. P-values are calculated by Mann Whitney U test. Values shown are background subtracted.


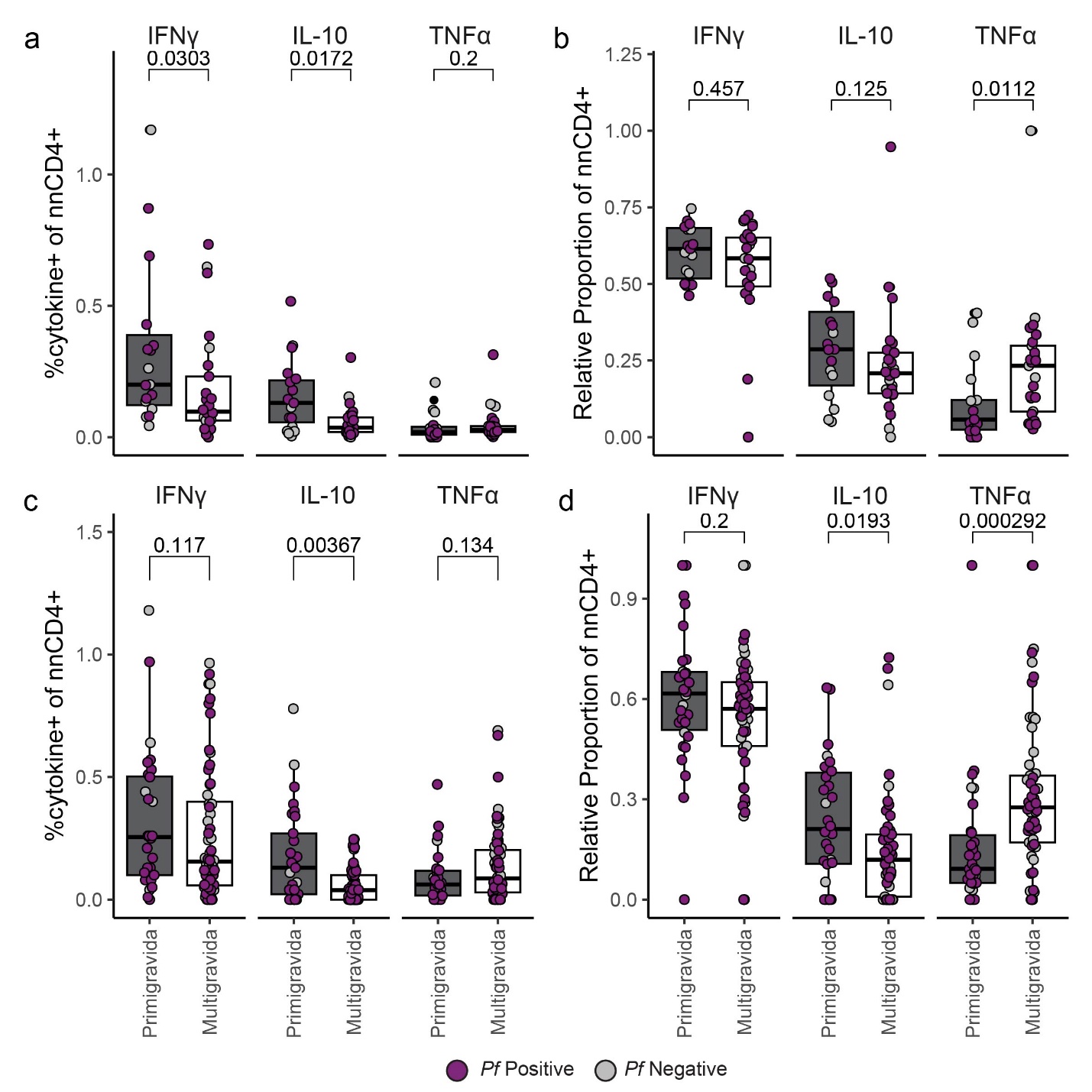


## Supplemental Figure 10: Percentage and Relative Proportion of nnCD4^+^ T cells expressing cytokines in the DPSP and PROMOTE cohort. (A and C) Percentage and (B and D) the relative proportion of nnCD4^+^ producing IFN​​γ, IL-10, and TNFα of total cytokine response after a 24 hour stimulation with iRBC from primigravid (DPSP (A and B) n=17, PROMOTE (C and D) n=30, dark grey) and multigravid (DPSP (A and B) n=25, PROMOTE (C and D) n=60, white) (purple = *Pf* qPCR positive, grey = *Pf* qPCR negative) pregnant women in DPSP are shown. Analysed by flow cytometry. Data shown are background (Media) subtracted. P-values are calculated by Mann Whitney U test.


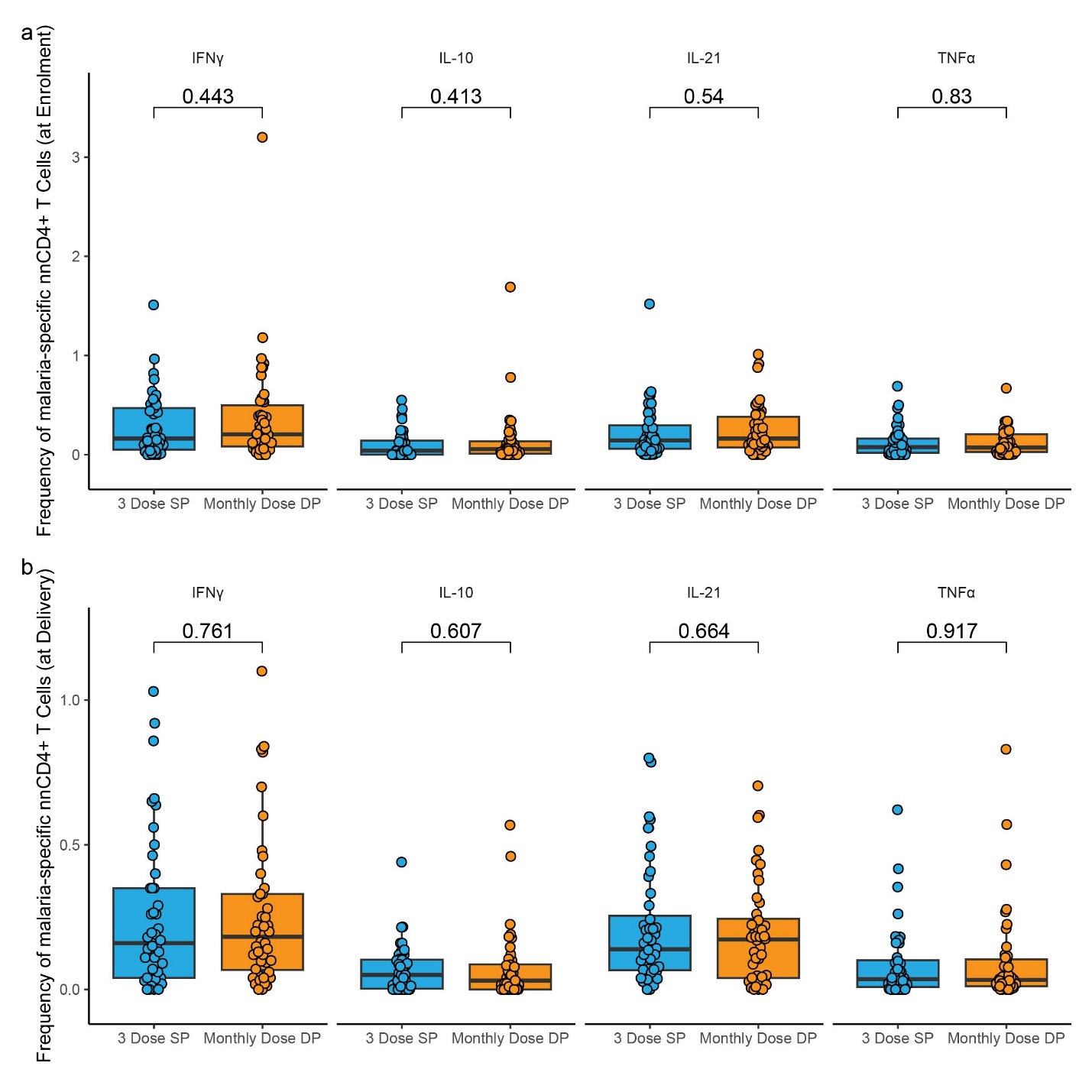


## Supplemental Figure 11: Comparison of cytokine producing CD4^+^ T cells at enrolment and delivery between IPTp arms among women in the PROMOTE clinical trial. Shown are percentage of malaria-specific, cytokine producing CD4^+^ T cells at enrolment (a) and delivery (b) among women randomized to receive IPTp with SP (given 3 doses, n=44, blue) or IPTp with DP (given monthly, n=44, orange). Data shown are background subtracted. P-values are calculated by Mann Whitney U test.


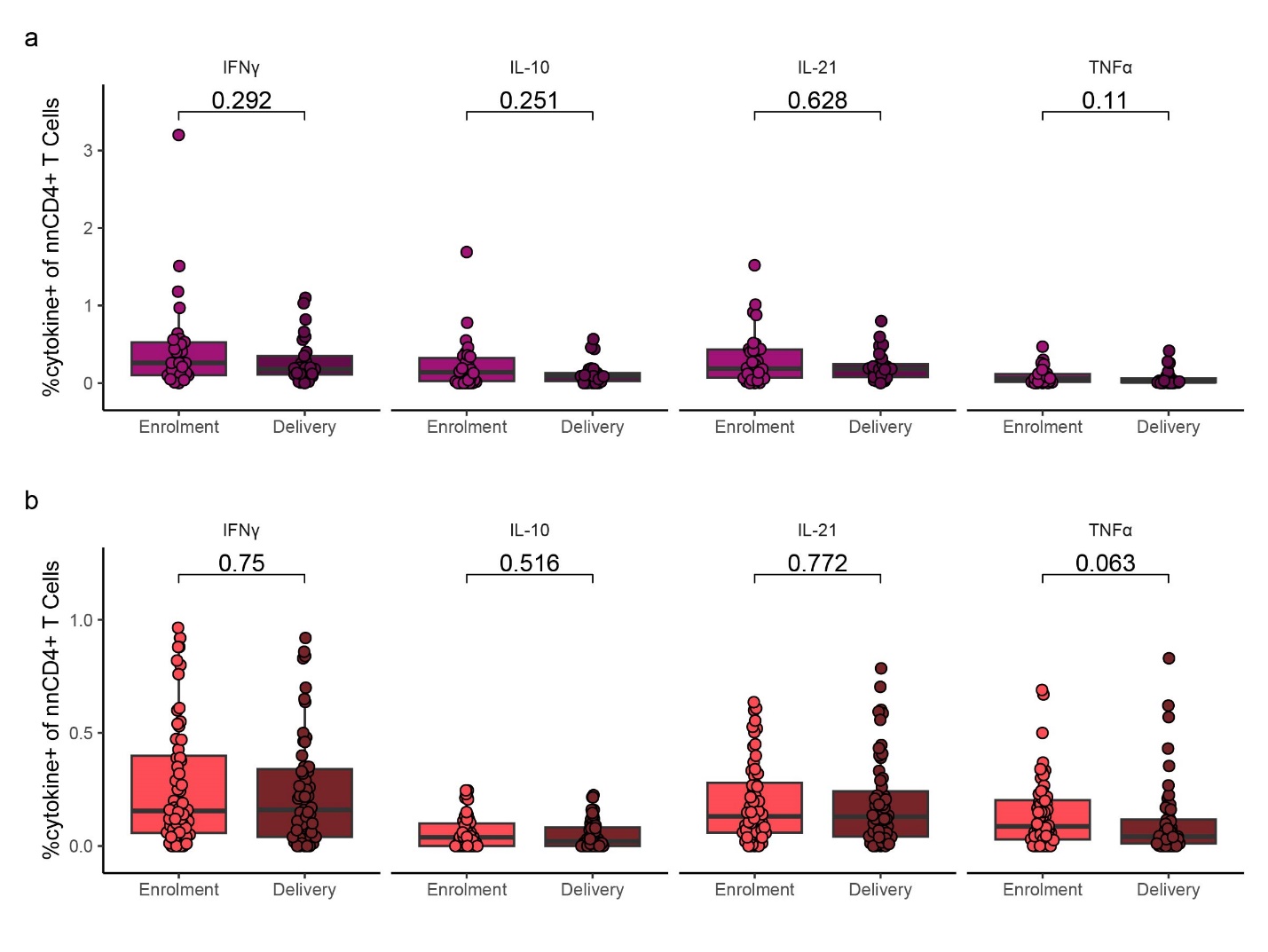


## Supplemental Figure 12: Comparison of T cell cytokine production at enrolment and delivery among primigravid and multigravid women. Shown are percentage of malaria-specific, cytokine producing nnCD4^+^ T cells at enrolment and delivery among (a) Primigravid women (n=30) and (b) Multigravid women (n=60). Data shown are background subtracted. P-values are calculated by Wilcoxon rank sum test.


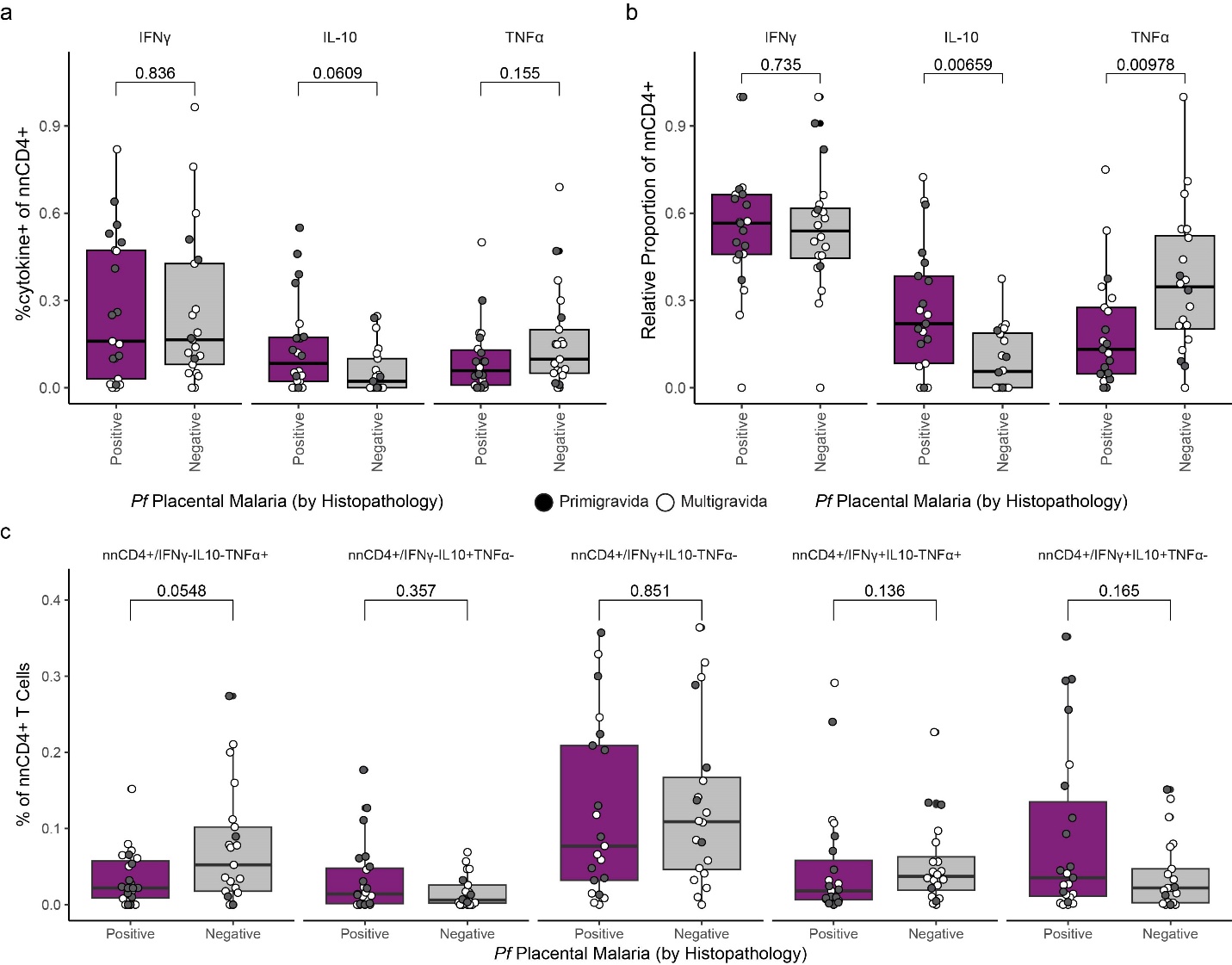


## Supplemental Figure 13: Comparison of nnCD4^+^ T cell subsets by placental malaria detected by histopathology. Shown are (a) percentage of T cells expressing cytokines (b) relative proportion of nnCD4^+^ T cells expressing cytokines), and (c) percentage of T cell Boolean gates by presence (n=22) or absence (n = 21) of PM as detected by histopathology among women randomized to IPTp with sulfadoxine pyrimethamine. Data shown are background subtracted. P-values are calculated by Mann Whitney U test.


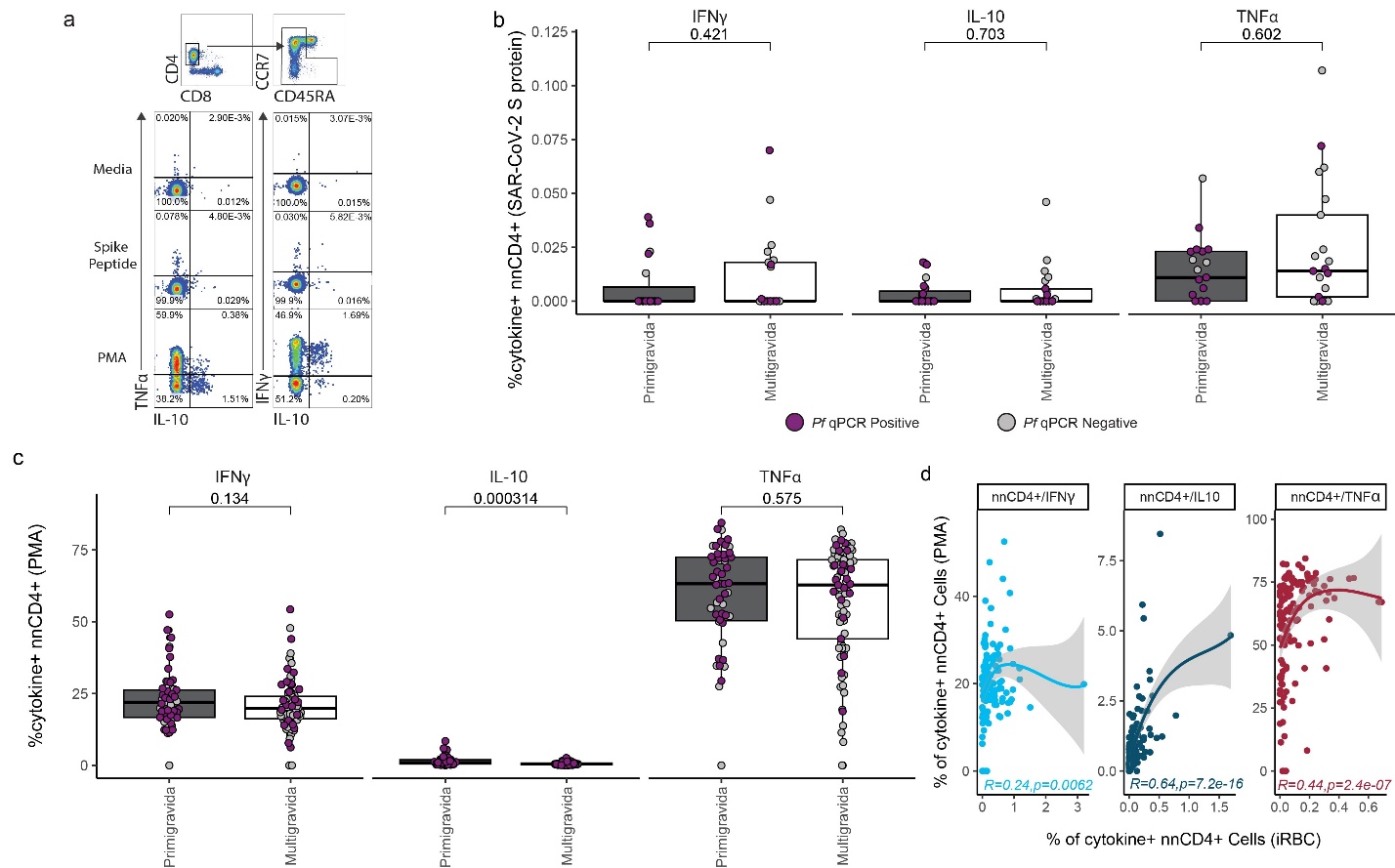


Supplemental Figure 14: nnCD4+ T cell response to SARS-CoV-2 Spike peptide and phorbol myristate acetate (PMA). Shown are (a) Dot plots of cytokine producing nnCD4+ T cells from DPSP cohort in response to SARS-CoV-2 Spike peptide and Phytohemagglutinin of a representative individual. (b) Depicted are nnCD4+ expressing IFN​​γ, IL-10, and TNFα in proportion of total cytokine response to SARS-CoV-2 spike peptide or (c) PMA from primigravida (n=50, dark grey) and multigravida (n=93, white) (purple = Pf qPCR positive, grey = Pf qPCR negative). (d) Loess regressions were fit to the percentage of nnCD4+ expression IFNγ (light blue), IL-10 (dark blue), and TNFΑ (dark red) iRBC stimulation by PMA stimulation (n=128). The P-values and R values are from Spearman correlations. Data shown are background subtracted. P-values are calculated by Mann Whitney U test.


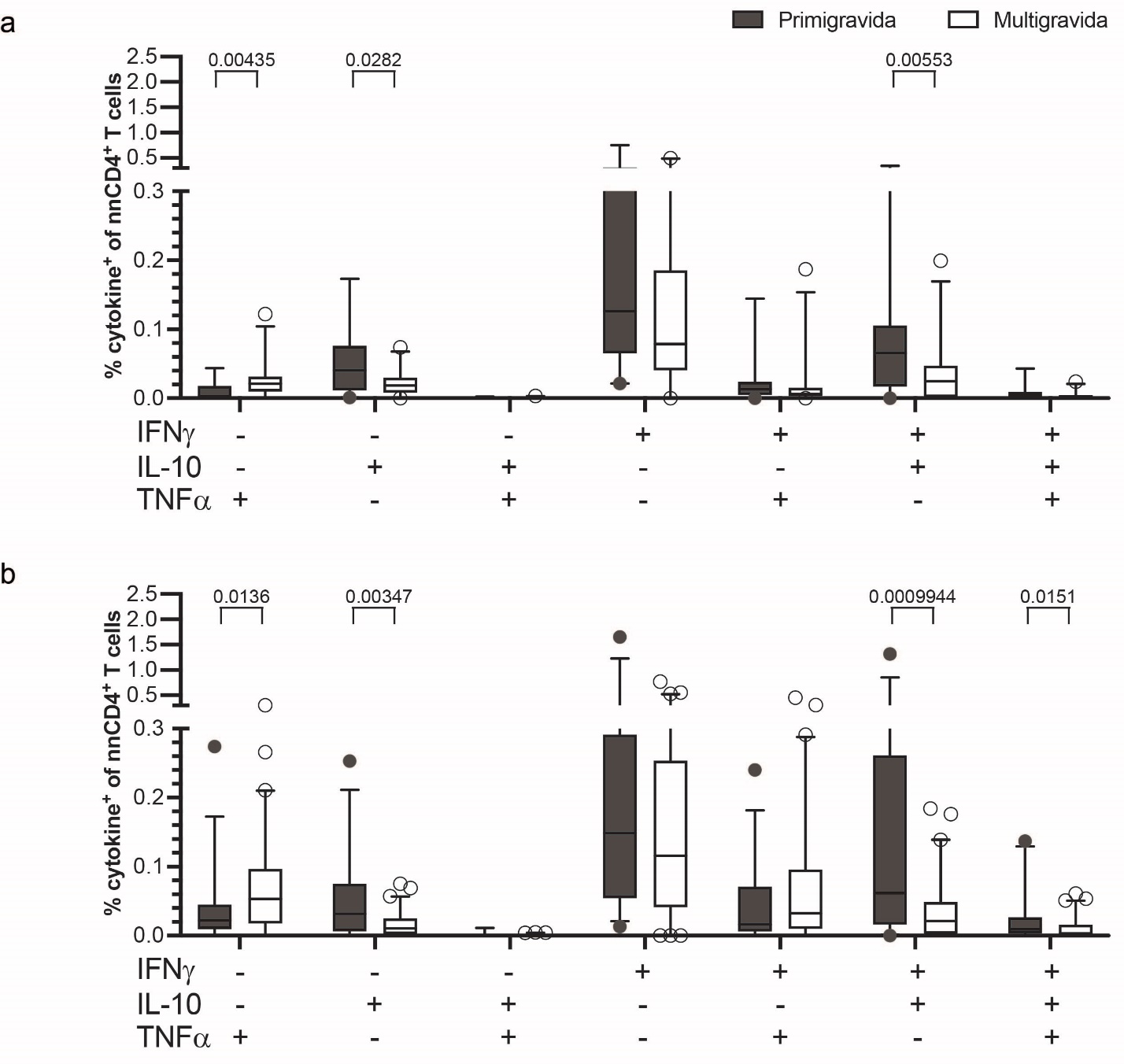


## Supplemental Figure 15: Comparison of nnCD4^+^ T cell cytokine producing subset between primigravid and multigravid women in DPSP and PROMOTE separately. Shown are percentage of malaria-specific, cytokine producing nnCD4^+^ T cell subsets at enrolment among (a) DPSP (primigravid women, n=19, dark grey, multigravid women, n=25, white) and (b) PROMOTE (primigravid women, n=30, dark grey, multigravid women, n=60, white). Data shown are background subtracted. P-values are calculated by Mann Whitney U test.


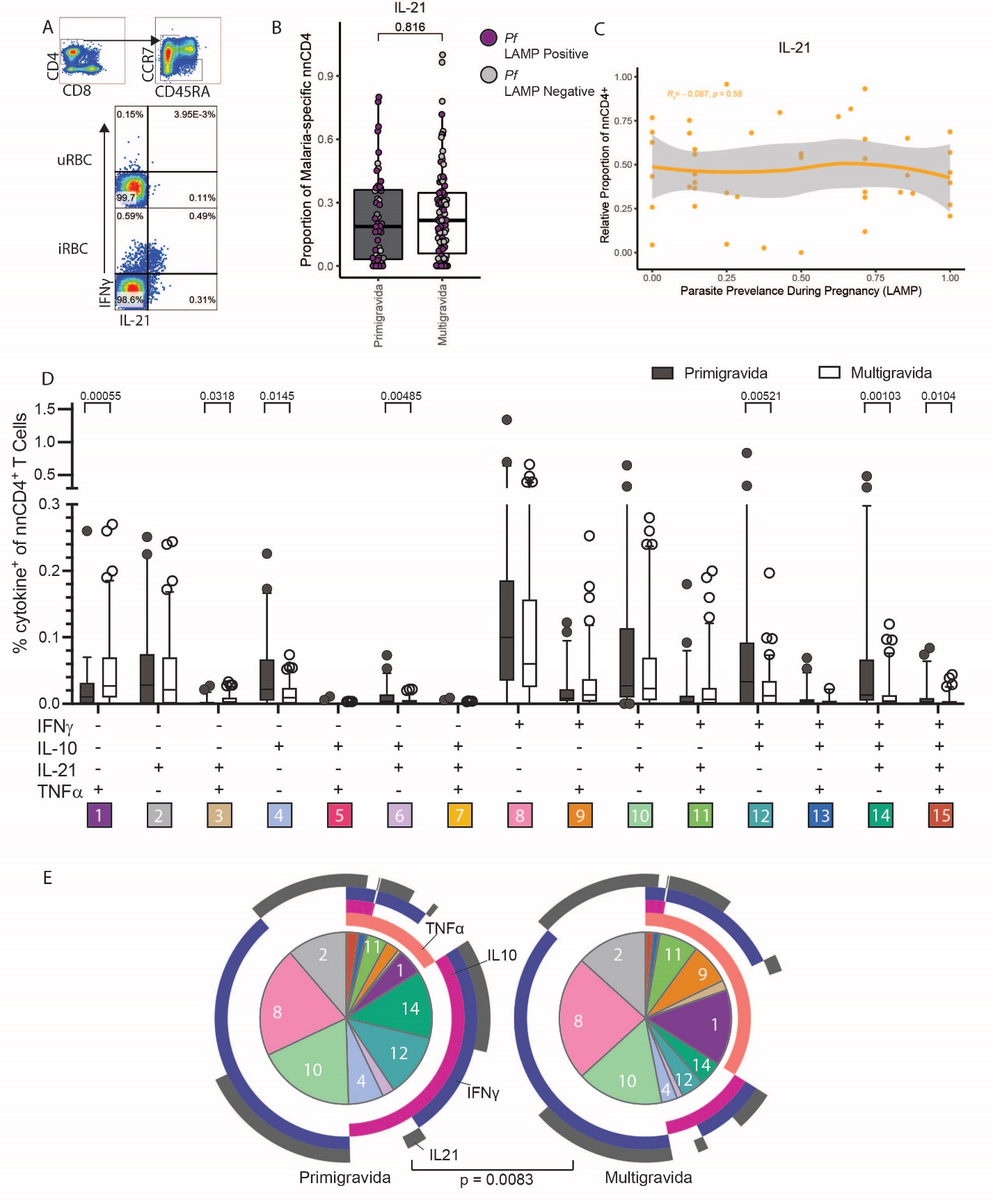


Supplemental Figure 16: nnCD4+ T cells subsets including IL-21. (a) Dot plot**s** of cytokine producing nnCD4^+^ T cells in response to iRBCs from a representative individual are depicted. (b) Shown are IL-21 cytokine producing nnCD4^+^ T cells in proportion to overall cytokine response between primigravid (n=49, dark grey) and multigravid (n=85, white) from both DPSP and PROMOTE. (purple = *Pf* LAMP positive, grey = *Pf* LAMP negative) p-values from Mann Whitney U test. (c) **T**he correlation of relative proportion of nnCD4^+^ T cells producing IL-21 with parasitaemia across pregnancy is depicted. P-value from spearman correlation (d and e) nnCD4^+^ T cell subsets including IL-21. P-value from (d) Mann Whitney U tests and (e) permutation test**.**

## Supplemental Table 1: Whole blood RNAseq quality control metrics

## Supplemental Table 2: List of genes differentially expressed in whole blood RNAseq among pregnant and non-pregnant women.

## Supplemental Table 3: Pathways upregulated in pregnant women.

## Supplemental Table 4: Differentially expressed genes in primigravida qPCR^+^

## Supplementary Table 5: Malaria-specific CD4^+^ T cell magnitude^1^ at enrolment^2^ and associations with gravidity

|  | **Primigravid** | **Multigravid** | **Univariable**^a^ | | **Multivariable**^b^ | |
| --- | --- | --- | --- | --- | --- | --- |
| **Malaria-specific CD4^+^ T cell population** | **Mean %, Log_10_(SD)** | **Mean %, Log_10_(SD)** | **Coef (95% CI)** | ***P*** | **Coef (95% CI)** | ***P*** |
| Any IFNγ^+^ | -0.60 (0.52) | -0.75 (0.49) | -0.15 (-0.38 – 0.08) | 0.20 |  |  |
| Any IL-10^+^ | -0.80 (0.50) | -1.25 (0.44) | -0.43 (-0.68- -0.18) | 0.001 | -0.32 (-0.65 - -0.002) | 0.049 |
| Any TNFα^+^ | -1.20 (0.46) | -1.05 (0.59) | 0.15 (-0.11- 0.41) | 0.26 |  |  |
| IFNγ^+^/IL-10^+^/TNFα^-^ | -1.19 (0.64) | -1.69 (0.61) | -0.49 (-0.78- -0.21) | 0.001 | -0.50 (-0.87 - -0.12) | 0.01 |
| IFNγ^-^/IL-10^+^/TNFα^-^ | -1.66 (0.72) | -2.10 (0.62) | -0.44 (-0.73- -0.15) | 0.003 | -0.31 (-0.68 – 0.07) | 0.11 |
| IFNγ^+^/IL-10^-^/TNFα^-^ | -0.86 (0.48) | -0.98 (0.52) | -0.12 (-0.35-0.10) | 0.29 |  |  |
| IFNγ^+^/IL-10^-^/TNFα^+^ | -1.66 (0.55) | -1.46 (0.55) | 0.20 (-0.05-0.46) | 0.11 |  |  |
| IFNγ^-^/IL-10^-^/TNFα^+^ | -1.57 (0.41) | -1.31 (0.47) | 0.26 (0.04-0.48) | 0.02 | 0.32 (0.04-0.60) | 0.027 |

^1^ CD4^+^ T cell magnitude defined as log_10_ % cytokine-producing cells among the total non-naïve CD4^+^ T cell population.

^2^ Enrolment visit during second trimester (12-20 weeks gestational age)

^a^ Associations measured using linear regression, modelling gravidity (exposure) with log_10_ % CD4 population among total non-naïve CD4^+^ T cells (outcomes)

^b^ Multivariable linear models adjusted for parasitaemia (measured by LAMP) at the time of measurement and maternal age at enrolment

## Supplementary Table 6: Malaria-specific CD4^+^ T cell proportions^1^ at enrolment^2^ and associations with gravidity

|  | **Primigravid** | **Multigravid** | **Univariable**^a^ | | **Multivariable**^b^ | |
| --- | --- | --- | --- | --- | --- | --- |
| **Malaria-specific CD4^+^ T cell population** | **Mean Proportion % (SD)** | **Mean Proportion % (SD)** | **Coef (95% CI)** | ***P*** | **Coef (95% CI)** | ***P*** |
| Any IFNγ^+^ | 62.8 (22.4) | 53.6 (24.4) | -9.2 (-19.83 – 1.43) | 0.09 |  |  |
| Any IL-10^+^ | 28.4 (23.2) | 14.1 (14.8) | -14.30 (-22.39 - -6.20) | 0.001 | -8.90 (-19.21 – 1.42) | 0.09 |
| Any TNFα^+^ | 14.7 (14.1) | 30.2 (20.1) | 15.54 (7.37 – 21.72) | <0.001 | 18.26 (7.58- 28.94) | 0.001 |
| IFNγ^+^/IL-10^+^/TNFα^-^ | 19.0 (13.9) | 8.6 (7.9) | -10.45 (-15.08 - -5.83) | <0.001 | -9.09 (-15.13 - -3.06) | 0.004 |
| IFNγ^-^/IL-10^+^/TNFα^-^ | 10.0 (12.5) | 6.1 (9.7) | -3.91 (-8.71 – 0.87) | 0.11 |  |  |
| IFNγ^+^/IL-10^-^/TNFα^-^ | 39.1 (18.5) | 36.0 (15.8) | -3.12 (-10.62 - 4.38) | 0.41 |  |  |
| IFNγ^+^/IL-10^-^/TNFα^+^ | 6.3 (5.7) | 13.0 (9.7) | 6.64 (2.80 - 10.47) | 0.001 | 6.61 (1.67-11.55) | 0.009 |
| IFNγ^-^/IL-10^-^/TNFα^+^ | 7.8 (9.8) | 16.2 (12.7) | 8.41 (3.14 - 13.69) | 0.002 | 11.13 (4.23-18.03) | 0.002 |

^1^ CD4^+^ T cell proportion defined as % CD4 population of total cytokine-producing CD4^+^ T cells

^2^ Enrolment visit during second trimester (12-20 weeks gestational age)

^a^Associations measured using linear regression, modelling gravidity (exposure) with % CD4 population as proportion of total cytokine-producing CD4^+^ T cells (outcomes)

^b^Multivariable linear models adjusted for parasitaemia (measured by LAMP) at the time of measurement and maternal age at enrolment.

## Supplementary Table 7: Malaria-specific CD4^+^ T cell proportions^1^ at enrolment^2^ and associations with *Pf* parasite prevalence in pregnancy

##

|  | **Univariable**^a^ | | **Multivariable**^b^ | |
| --- | --- | --- | --- | --- |
| **Malaria-specific CD4^+^ T cell population** | **Coef (95% CI)** | ***P*** | **Coef (95% CI)** | ***P*** |
| Any IFNγ^+^ | 0.04 (-0.38-0.47) | 0.84 |  |  |
| Any IL-10^+^ | 1.01 (0.58-1.45) | <0.001 | 0.64 (0.18-1.10) | 0.008 |
| Any TNFα^+^ | -0.55 (-1.04 - -0.06) | 0.027 | -0.32 (-0.74 – 0.11) | 0.14 |
| IFNγ^+^/IL-10^+^/TNFα^-^ | 1.45 (0.76-2.15) | <0.001 | 0.82 (0.07 – 1.56) | 0.032 |
| IFNγ^-^/IL-10^+^/TNFα^-^ | 1.50 (0.46-2.54) | 0.006 | 1.01 (0.12-1.90) | 0.027 |
| IFNγ^+^/IL-10^-^/TNFα^-^ | -0.30 (-1.0 - 0.39) | 0.38 |  |  |
| IFNγ^+^/IL-10^-^/TNFα^+^ | -1.33 (-2.29 - -0.38) | 0.007 | -0.86 (-1.69- -0.02) | 0.046 |
| IFNγ^-^/IL-10^-^/TNFα^+^ | -0.48 (-1.29 – 0.34) | 0.25 |  |  |

^1^ CD4^+^ T cell proportion defined as % CD4 population of total cytokine-producing CD4^+^ T cells.

^2^ Enrolment visit during second trimester (12-20 weeks gestational age) among n=44 women randomized to receive intermittent preventive treatment with sulfadoxine-pyrimethamine

^a^ Associations measured using linear regression, modelling proportion of CD4^+^ T cells (exposure) with subsequent Pf parasite prevalence measured at monthly visits during pregnancy by Loop-mediated isothermal amplification (LAMP)

^b^ Multivariable linear models adjusted for parasitaemia status at the time of measurement (by LAMP), maternal age at enrolment, and gravidity (dichotomous)

## Supplementary Table 8: Malaria-specific CD4^+^ T cell magnitude^1^ at enrolment^2^ and associations with *Pf* parasite prevalence in pregnancy

|  | **Univariable**^a^ | | **Multivariable**^b^ | |
| --- | --- | --- | --- | --- |
| **Malaria-specific CD4^+^ T cell population** | **Coef (95% CI)** | ***P*** | **Coef (95% CI)** | ***P*** |
| Any IFNγ^+^ | 0.04 (-0.17 – 0.25) | 0.69 |  |  |
| Any IL-10^+^ | 0.26 (0.09 – 0.42) | 0.003 | 0.12 (-0.05 – 0.28) | 0.15 |
| Any TNFα^+^ | -0.19 (-0.38 - -0.002) | 0.048 | -0.16 (-0.32 – 0.002) | 0.05 |
| IFNγ^+^/IL-10^+^/TNFα^-^ | 0.22 (0.06 – 0.39) | 0.009 | 0.06 (-0.11 – 0.22) | 0.48 |
| IFNγ^-^/IL-10^+^/TNFα^-^ | 0.20 (0.06 – 0.34) | 0.006 | 0.10 (-0.02 – 0.23) | 0.16 |
| IFNγ^+^/IL-10^-^/TNFα^-^ | -0.06 (-0.26 – 0.15) | 0.57 |  |  |
| IFNγ^+^/IL-10^-^/TNFα^+^ | -0.14 (-0.32 – 0.03) | 0.11 |  |  |
| IFNγ^-^/IL-10^-^/TNFα^+^ | -0.32 (-0.61 - -0.03) | 0.032 | -0.16 (-0.42 – 0.098) | 0.22 |

^1^ CD4^+^ T cell magnitude defined as log_10_ % cytokine-producing cells among the total non-naïve CD4^+^ T cell population.

^2^ Enrolment visit during second trimester (12-20 weeks gestational age) among n=44 women randomized to receive intermittent preventive treatment with sulfadoxine-pyrimethamine

^a^Associations measured using linear regression, modelling log_10_ % CD4 population among total non-naïve CD4^+^ T cells (exposure) with subsequent Pf parasite prevalence measured at monthly visits during pregnancy by Loop-mediated isothermal amplification (LAMP)

^b^Multivariable linear models adjusted for parasitaemia status at the time of measurement (by LAMP), maternal age at enrolment, and gravidity (dichotomous)

##

## Supplementary Table 9: Malaria-specific CD4^+^ T cell proportions^1^ at enrolment^2^ and associations with placental malaria measured by *Plasmodium falciparum* detection by Loop-mediated isothermal amplification (LAMP) at delivery.

|  | **No Placental Malaria (n=37)** | **Placental Malaria (n=6)** | **Univariate^a^** | | **Multivariate^b^** | |
| --- | --- | --- | --- | --- | --- | --- |
| **Malaria-specific CD4^+^ T cell population** | **Mean Proportion % (SD)** | **Mean Proportion % (SD)** | **OR (95% CI)** | ***P*** | **OR (95% CI)** | ***P*** |
| Any IFNγ^+^ | 52.0 (27.2) | 67.3 (15.0) | 1.03 (0.98-1.08) | 0.21 |  |  |
| Any IL-10^+^ | 14.8 (17.1) | 46.7 (20.7) | 1.07 (1.02 – 1.13) | 0.005 | 1.06 (1.01-1.13) | 0.035 |
| Any TNFα^+^ | 30.0 (22.2) | 10.4 (8.6) | 0.94 (0.87 – 1.00) | 0.07 |  |  |
| IFNγ^+^/IL-10^+^/TNFα^-^ | 10.7 (11.4) | 28.6 (16.0) | 1.09 (1.02 – 1.16) | 0.01 | 1.06 (0.99 – 1.14) | 0.09 |
| IFNγ^-^/IL-10^+^/TNFα^-^ | 5.1 (6.6) | 16.2 (19.0) | 1.09 (1.0-1.20) | 0.06 |  |  |
| IFNγ^+^/IL-10^-^/TNFα^-^ | 32.5 (16.7) | 32.8 (13.5) | 1.0 (0.95 – 1.06) | 0.97 |  |  |
| IFNγ^+^/IL-10^-^/TNFα^+^ | 13.9 (10.9) | 4.0 (3.8) | 0.80 (0.64 – 0.998) | 0.048 | 0.74 (0.55 – 0.99) | 0.046 |
| IFNγ^-^/IL-10^-^/TNFα^+^ | 15.3 (14.0) | 5.5 (4.9) | 0.91 (0.80 – 1.03) | 0.12 |  |  |

^1^ CD4^+^ T cell proportion defined as % CD4 population of total cytokine-producing CD4^+^ T cells.

^2^ Enrolment visit during second trimester (12-20 weeks gestational age) among n=43 women randomized to receive intermittent preventive treatment with sulfadoxine-pyrimethamine and with placental *Plasmodium falciparum* results measured by LAMP at delivery

^a^ Associations measured using logistic regression, modelling proportion of CD4^+^ T cells (exposure) with placental malaria as measured by LAMP (outcome)

^b^ Multivariate logistic models adjusted for parasitaemia (measured by LAMP) at the time of measurement, maternal age at enrolment, and gravidity (dichotomous)

## Supplementary Table 10: Malaria-specific CD4^+^ T cell magnitude^1^ at enrolment^2^ and associations with placental malaria measured by *Plasmodium falciparum* detection by Loop-mediated isothermal amplification (LAMP) at delivery.

|  | **No Placental Malaria (n=37)** | **Placental Malaria (n=6)** | **Univariate^a^** | | **Multivariate^b^** | |
| --- | --- | --- | --- | --- | --- | --- |
| **Malaria-specific CD4^+^ T cell population** | **Mean %, Log_10_(SD)** | **Mean %, Log_10_(SD)** | **OR (95% CI)** | ***P*** | **OR (95% CI)** | ***P*** |
| Any IFNγ^+^ | -0.78 (0.57) | -0.53 (0.26) | 2.72 (0.39 – 19.20) | 0.32 |  |  |
| Any IL-10^+^ | -1.44 (0.57) | -0.72 (0.18) | 16.5 (1.48 – 184.46) | 0.02 | 10.2 (0.63 – 166.24) | 0.10 |
| Any TNFα^+^ | -1.05 (0.60) | -1.30 (0.44) | 0.51 (0.12 – 2.23) | 0.37 |  |  |
| IFNγ^+^/IL-10^+^/TNFα^-^ | -1.83 (0.80) | -0.95 (0.31) | 9.55 (1.20 – 75.82) | 0.03 | 5.28 (0.56 – 50.21) | 0.15 |
| IFNγ^-^/IL-10^+^/TNFα^-^ | -2.14 (0.70) | -1.39 (0.43) | 8.03 (1.13 – 57.23) | 0.038 | 7.73 (0.61 – 97.83) | 0.11 |
| IFNγ^+^/IL-10^-^/TNFα^-^ | -1.04 (0.57) | -0.87 (0.36) | 1.92 (0.31 – 11.80) | 0.48 |  |  |
| IFNγ^+^/IL-10^-^/TNFα^+^ | -1.48 (0.61) | -1.75 (0.39) | 0.45 (0.09 – 2.36) | 0.35 |  |  |
| IFNγ^-^/IL-10^-^/TNFα^+^ | -1.30 (0.40) | -1.58 (0.15) | 0.13 (0.01 – 2.09) | 0.15 |  |  |

^1^ CD4^+^ T cell magnitude defined as log_10_ % cytokine-producing cells among the total non-naïve CD4^+^ T cell population.

^2^ Enrolment visit during second trimester (12-20 weeks gestational age) among n=43 women randomized to receive intermittent preventive treatment with sulfadoxine-pyrimethamine and with placental *Plasmodium falciparum* results measured by LAMP at delivery

^a^Associations measured using logistic regression, modelling log_10_ % CD4 population among total non-naïve CD4^+^ T cells (exposure) with placental malaria as measured by LAMP (outcome)

^b^Multivariate logistic models adjusted for parasitaemia (measured by LAMP) at the time of measurement, maternal age at enrolment, and gravidity (dichotomous)

## Supplemental Table 11: Cytokine capture Cell counts

| **IFNγ^+^IL-10^+^TNFα^-^ (Number of cells)** | **IFNγ^-^IL-10^-^TNFα^+^ (Number of Cells)** |
| --- | --- |
| **299** | **1317** |
| **211** | **1381** |
| **280** | **3174** |

## Supplemental Table 12: Differentially expressed genes from sorted cells.
